# Data-Driven Electrochemistry Reveals the Impact of Hydrophobicity on Aptamer Cross-Reactivity

**DOI:** 10.1021/acsmeasuresciau.6c00040

**Published:** 2026-05-20

**Authors:** Emily Carroll, Michael A. Pence, Elizabeth Winterholler, Taylor D. Sparks, Shelley D. Minteer

**Affiliations:** † Department of Chemistry, 7060 University of Utah, Salt Lake City, Utah 84112, United States; ‡ Kummer Institute Center for Resource Sustainability, 14589 Missouri University of Science and Technology, Rolla, Missouri 65409, United States; § Department of Materials Science & Engineering, 7060 University of Utah, Salt Lake City, Utah 84112, United States

**Keywords:** aptamers, biosensing, electrochemical sensors, progesterone, automation, machine learning

## Abstract

Electrochemical aptamer-based (E-AB) biosensors offer a promising platform for reagentless detection of molecular targets, yet aptamer recognition can be limited by cross-reactivity, particularly for hydrophobic analytes such as steroid hormones. To investigate how cross-reactivity influences E-AB sensor performance, we use automation and machine learning to screen a library of possible interferent molecules against a steroid-binding aptamer, with progesterone serving as a physiologically relevant test case. Here, we develop a label-free E-AB sensor for progesterone detection using a methylene blue-modified aptamer anchored with a hexanethiol linker. We then used an automated electrochemistry platform to perform reproducible and high-throughput characterization of our sensor through titration and frequency mapping experiments, identifying optimal frequencies for square-wave voltammetry interrogation. Our automated platform improved experimental throughput by 3-fold that of manual experimentation and greatly improved reproducibility when characterizing our aptamer-modified electrode. Over the course of this work, we collected 20,000 voltammograms demonstrating the high-throughput capability of our platform. To evaluate the specificity of the aptamer sensor, we used our automated platform to screen an interferent scope of 40 structurally and functionally diverse molecules. We used interpretable machine learning to better understand the chemical characteristics of interferent molecules that resulted in sensor cross-reactivity, identifying hydrophobicity as a key molecular descriptor in predicting the aptamer response. We found that the aptamer was cross-reactive toward molecules with hydrophobicities similar to progesterone, irrespective of molecular structure. This cross-reactivity insight is an important consideration for the counterselection process during aptamer design.

## Graphical abstract

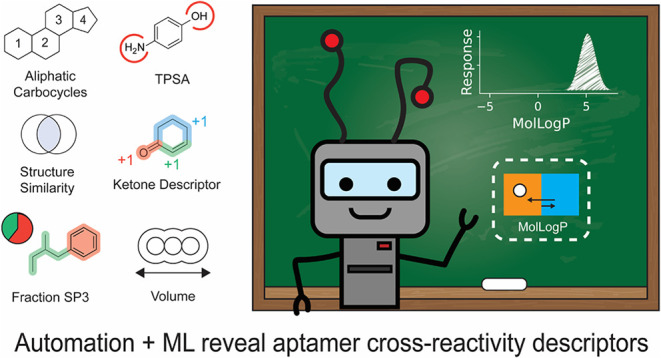

## Introduction

Personal health monitoring has become essential for understanding dynamic physiological changes, enabling real-time, point-of-care therapeutics.^1^ To realize personal health monitoring, there is a need for highly sensitive and selective biosensors capable of continuous monitoring. Electrochemical platforms are well suited for continuous monitoring as the instrumentation is portable and can be used for point-of-care applications and has been utilized extensively for monitoring metabolites and neurotransmitters *in vivo*.^2–6^ The primary drawback of electrochemistry is that it requires redox-active analytes, and it lacks the chemical specificity of other analytical methods. Modification of electrodes with biorecognition elements circumvents these drawbacks and enables selective detection of non-redox active targets. Aptamers (short, single-stranded DNA or RNA oligonucleotides) have emerged as promising biorecognition elements in the development of electrochemical-based sensors due to their high affinities, stability, *in vitro* selection, and low cost compared to proteins and immunoassays.^7–10^ Additionally, they can be modified with redox tags, fluorophores, and moieties for attachment to surfaces.^11^

Electrochemical aptamer-based (E-AB) biosensors have shown promise as a platform for reagentless detection of molecular and biological analytes.^12,13^ E-AB sensors measure variations in electrochemical response resulting from binding-induced changes in aptamer conformation.^14–16^ The electrochemical response can come from either a redox tag attached to the aptamer or a freely diffusing redox mediator in solution. A conformational change of the aptamer upon target binding changes electron transfer kinetics and, consequently, the Faradaic response.^17,18^ Despite the promise of aptamers as recognition units in electrochemical sensors, they can suffer from cross-reactivity due to their affinity-based sensing mechanism, especially when aptamer sequences are developed for hydrophobic targets.^19,20^ When compared with other biomolecules such as proteins, aptamers can struggle with forming hydrophobic cavities. Because of this, aptamers with hydrophobic targets typically have moderate binding affinities and substantial cross-reactivity.^19,21^ Strategies have been explored to address this limitation, including the incorporation of hydrophobic or otherwise modified nucleobases during aptamer selection. Because natural nucleic acids have limited chemical diversity and primarily engage targets through polar and electrostatic interactions, the introduction of noncanonical nucleotides containing side chains with hydrophobic functionality can enhance binding to hydrophobic targets.^22–24^ Steroids are a key class of hydrophobic molecules that regulate physiological functions and biological signaling pathways in the body, often found in lipophilic environments.^21,25^ The hydrophobic nature of steroids makes it difficult to sense them with affinity-based mechanisms such as aptamers.^21^ It follows that the ubiquitous nature of steroid molecules in human health, along with their associated sensing challenges, makes them an ideal test case for studying cross-reactivity in E-AB sensors.

In this work, we investigate progesterone (P4), a naturally occurring endogenous steroid hormone produced by the body.^26^ Progesterone expression fluctuates throughout the reproductive cycle, regulating ovulation, and is essential in supporting early pregnancy.^27^ Globally, millions of women suffer from infertility and miscarriage, and simple tracking of progesterone levels during ovulation can increase fertility odds.^28,29^ Current hormone testing relies on serum collected in clinical settings and analyzed by commercial laboratories, with results provided in a few days to a week.^29^ In order to accurately capture the fluctuations in hormone levels, continuous monitoring is needed. Electrochemical detection of progesterone has been accomplished using E-AB sensors, but existing sensor scaffolds do not permit continuous monitoring. Previous work by Jiménez and coworkers identified an aptamer sequence with high binding affinity to progesterone for use in an impedimetric sensor,^30^ and a follow-up study by the same group demonstrated a truncated sequence that had a 16 times smaller dissociation constant than that of the nontruncated aptamer (2.1 nM, compared to 35 nM).^31^ A similar effort by Kumar and coworkers developed an impedimetric E-AB sensor with extended stability in milk and serum.^32^ While these impedimetric sensors provided high affinity to progesterone, they required the addition of an exogenous redox mediator to the sample, precluding them from use in continuous monitoring.

In this work, we systematically screen a large library of possible interferent molecules against a steroid-binding aptamer to understand how cross-reactivity shapes the performance and interpretability of E-AB sensors. We aim to identify key interferents that will help guide future aptamer counterselection strategies, which have been shown to improve aptamer selectivity to hydrophobic targets.^21^ As a test case, we use progesterone to provide a representative and relevant benchmark for evaluating steroidal cross-reactivity. We developed a label-free E-AB sensor for progesterone detection by modifying the truncated oligonucleotide sequence developed by Alhadrami and coworkers^31^ with a methylene blue redox tag and a hexanethiol group for self-assembly onto a gold electrode. We then used an automated platform based on eLab^33^ and Hard Potato^34^ to perform reproducible and high-throughput characterization of our E-AB sensor. Our automated platform allowed for a comprehensive screen of 40 structurally and functionally diverse interferent molecules, which would be prohibitively time-consuming if carried out manually. We used interpretable machine learning to better understand what chemical characteristics of interferent molecules resulted in sensor cross-reactivity, identifying the computed water-octanol partition coefficient as a key molecular descriptor in predicting aptamer response (Figure 1).

**Figure 1:**
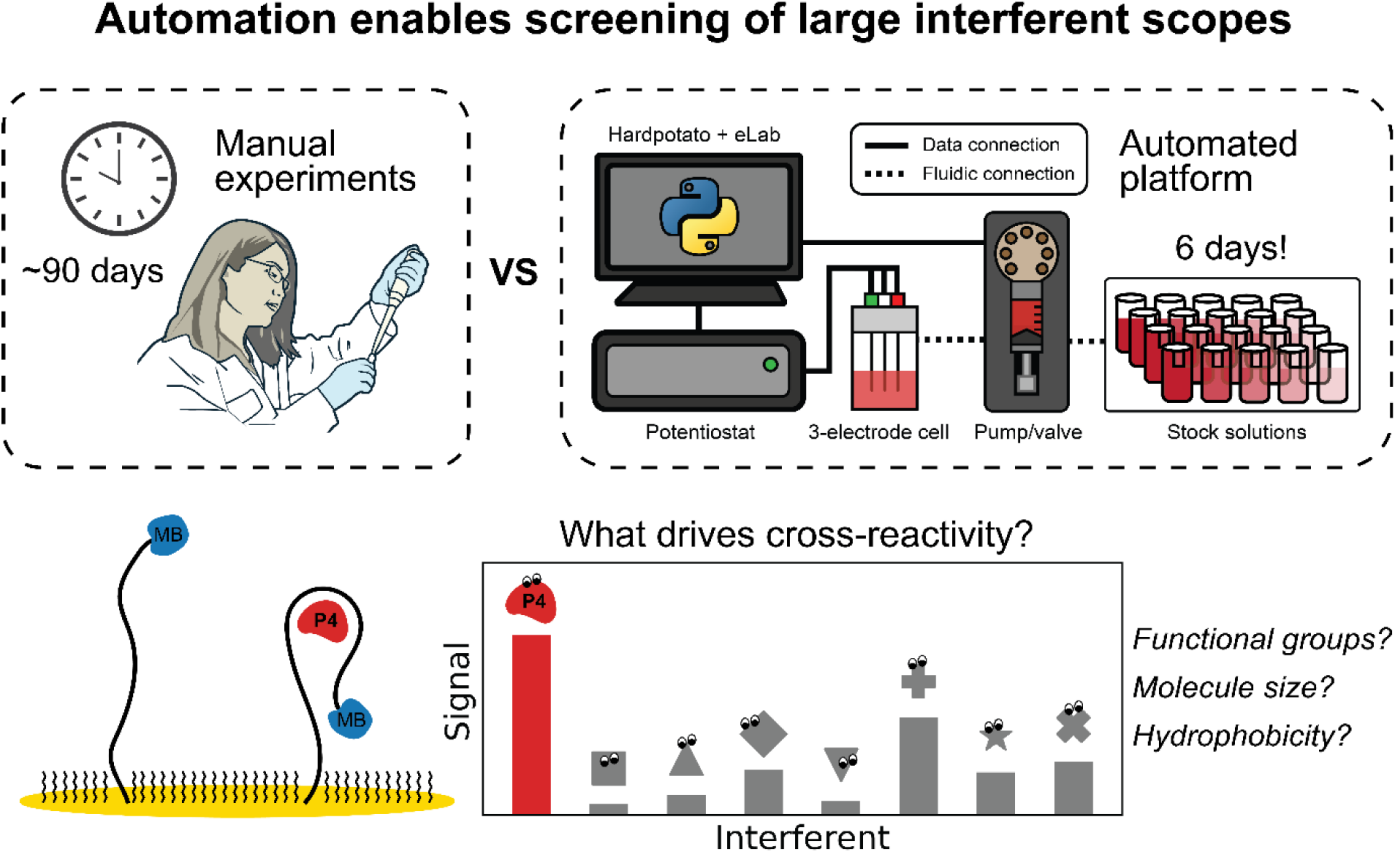
A label-free electrochemical aptamer-based (E-AB) sensor for progesterone detection was developed using a methylene-blue (MB)-modified aptamer sequence, self-assembled on a gold electrode. Automated electrochemistry platforms eLab and Hard Potato were used to perform reproducible and high-throughput sensor characterization and to evaluate the sensor’s cross-reactivity.

## Methods

### Modified DNA Oligos

Oligonucleotide sequences were purchased from Integrated DNA Technologies, Inc. (https://www.idtdna.com) and included a 5′ thiol modifier and a 3′ methylene blue modifier. The 5′ thiol modifier is attached to the oligo via a C6 connection with a disulfide bond. Once the disulfide bond is cleaved by a chemical reducing agent, it gives a free sulfur group to assemble to the gold electrode, with the oligo attached on a C6 linker. This C6 linker is convenient when using SAM blocking layers of equal length (i.e., mercaptohexanol or hexanethiol). Notably, when the disulfide bond is cleaved, the group on the other side of the oligo is mercaptohexanol, which can bind to the electrode surface, albeit at much lower concentrations than the SAM blocking layer. The UNAFold web server was used to predict the secondary structure of the aptamer (Figure S1).^35^ We used the following modification codes and sequences when requesting our modified aptamer from the supplier:

Progesterone aptamer sequence (25-mer): GATTAACATTAGCCCACCGCCCACC

5′ modification code: /5ThioMC6-D/

3′ modification code:/3MeBlN/

Overall aptamer sequence with modifications: /5ThioMC6-D/GATTAACATTAGCCCACCGCCCACC/3MeBlN/

The modified DNA oligo is shipped as a lyophilized powder and was resuspended in an aqueous solution and stored at −20 °C. We resuspended the oligo in TE buffer (10 mM Tris, 0.1 mM EDTA, pH 8.0) to help maintain a constant pH and prevent nuclease digestion of the DNA. We recommend diluting the oligo to a 50 or 100 μM stock concentration. Once resuspended, the oligo should be stored at −20 °C, where it is stable for over 24 months.^36^ Methylene blue is light sensitive so the oligo vial should be wrapped in foil for additional protection against light.^37^

### Materials

6-Mercapto-1-hexanol (MCH), 1-hexanethiol (HxSH), and tris­(2-carboxyethyl)­phosphine hydrochloride (TCEP) were purchased from Sigma-Aldrich (St. Louis, MO). Binding buffer (50 mM Tris (pH 7.5), 150 mM NaCl, 5 mM MgCl_2_) was prepared from chemicals purchased from Sigma-Aldrich (St. Louis, MO). Sulfuric acid (H_2_SO_4_), sodium hydroxide (NaOH), and methanol (certified ACS) were purchased from Fisher Scientific. Progesterone, d-phenylalanine, urea, cholesterol, dodecanoic acid, formic acid, glucose, cortisol, aspirin, l-glutamic acid, retinol, estradiol, acetaminophen, pregnenolone, cholic acid, malonic acid, melatonin, niacin, *N*-acetylglutamine, caffeine, L-carnitine, 4-aminophenol, betaine, benzyl alcohol, 3-indoleacetic acid, ampicillin, cobalamin, 17-hydroxyprogesterone, NADH, hydroquinone, estriol, styrene, sucrose, thiamine, butyric acid, estrone, cholecalciferol, creatine, acetic acid, FAD, l-ascorbic acid, triclosan, linoleic acid, loratadine, and loperamide were purchased from Sigma-Aldrich (St. Louis, MO). We prepared all aqueous solutions using deionized water from a Milli-Q Direct purification system, with a resistivity of 18 MΩ. Progesterone and other water-insoluble interferent molecules were dissolved in 100% methanol to ensure complete solubilization and accurate stock concentration. Water-soluble interferents were dissolved in deionized water. A full list of preparation conditions is given in Table S1. In working solutions, the total concentration of methanol was kept constant at 5%. Gold working electrodes (CHI101, diameter 2 mm) and calomel reference electrodes (SCE, CHI150) were purchased from CH Instruments, Inc. (Austin, TX). For polishing electrodes, cloth pads (PN: 40-7212) and alumina slurry (0.05 μm deagglomerated alumina micropolish) were purchased from Buehler (Lake Bluff, IL). Oligonucleotides, modified on the 5′ end with hexanethiol and on the 3′ end with methylene blue were purchased from and HPLC-purified by Integrated DNA Technologies, Inc. (Coralville, IA). The structure of the oligonucleotide employed in this work (including modifications) is shown in Figure S2.

### Ensuring Interferent Diversity

We used dimensionality reduction to map relevant chemical space, and qualitatively ensure structural diversity in the interferent set.^38,39^ To limit the space of possible interferents we used the 3128 compounds included in the Human Metabolome Database that have been quantitatively detected in blood.^40^ We removed compounds with SMILES that were not accepted by RDKIT, were not organic (no C in the SMILES string), or belonged to complex lipid metabolites that would not be commercially available, resulting in 1421 relevant compounds. We then generated Morgan fingerprints (1024 bits, radius = 2) for all remaining compounds. Dimensionality reduction was performed by first reducing the 1024 dimension fingerprints to 160 dimensions (∼85% explained variance) using Principal Component Analysis (PCA), followed by t-distributed Stochastic Neighbor Embedding (t-SNE) to further reduce the data set to 2 dimensions (Figure S3). The chosen interferents were then selected based on cost and availability, ensuring that they were not localized in the same region of chemical space.

## Sensor Fabrication

### Preparation of Electrodes

Gold electrodes were prepared by mechanically polishing for 1 min with a 0.05 μM alumina slurry followed by sonication to remove polishing material. The electrode was electrochemically cleaned through reductive and oxidative cycling in basic and acidic conditions, respectively (Figure S4). First, in 0.5 M NaOH, the potential was swept from −0.3 V to −1.6 V vs SCE (scan rate, 0.5 V/s; sample interval, 0.01 V) for 200 cycles. Next, in 0.5 M H_2_SO_4_, the potential was swept from 0 to 1.6 V vs SCE (scan rate, 0.5 V/s; sample interval, 0.01 V) for 200 cycles. We then rinsed the electrode with deionized water.

### Immobilization of Aptamer on Electrode

Electrodes are prepared through a stepwise incubation procedure, where the aptamer is incubated first, followed by a backfilling step with hexanethiol to form a self-assembled monolayer.^41–43^ To prepare the aptamer solutions, we first chemically reduced 1 μL of 50 μM thiolated MB-modified aptamer with 1.5 μL of 5 mM TCEP in a microcentrifuge tube (2 h in the dark, at room temperature). We then diluted the chemically reduced aptamer to a final concentration of 150 nM using 330 μL of binding buffer (50 mM Tris (pH 7.5), 150 mM NaCl, 5 mM MgCl_2_). The freshly cleaned electrode was then placed into the diluted aptamer solution to incubate for 3 h. The electrode was then transferred to a freshly prepared 1 mM hexanethiol solution and incubated overnight. For experiments using a different concentration of aptamer, see Table S2 for preparation conditions. After sitting overnight, for a minimum of 12 h, the electrode was rinsed with deionized water and set to rest in binding buffer solution for ∼30 min to remove any unbound aptamer or thiol. The fabrication protocol used here was extensively optimized following established procedures for E-AB sensor preparation.^13,41–43^

We outline the following experimental notes that were key to successfully fabricating our E-AB sensor:

• The aptamer stock solution should be stored at −20 °C. Completely thaw the aptamer solution at room temperature and vortex or centrifuge it to homogenize solution before use.

• Prepare fresh TCEP and hexanethiol solutions immediately before use.

• When chemically reducing the aptamer, the solution color should change from blue to colorless. Sometimes, when using only 100× TCEP (as recommended by suppliers) or only letting the solution rest for 1 h, the solution can still be blue. For this reason, we used 150× TCEP and let the solution reduce for 2 h.

### Automated Electrochemical Measurements

We used a CH Instruments CHI660E potentiostat to perform all electrochemical measurements. We used a syringe pump and a fluidic switching valve (RUNZE SY-01B) for solution handling and an Arduino-based solenoid driver for gas flow handling. All instruments were connected to the computer and controlled through Python (ver. 3.13.2). We used modified versions of the Hard Potato and eLab libraries (HardPotato GitHub: https://github.com/mapence2/hardpotato; eLab GitHub: https://github.com/mapence2/elabAPI) to control the potentiostat and solution-handling hardware, respectively. A bill of materials, 3D printing files, and a schematic for the Arduino-based solenoid driver are available in the Supporting Information (Figure S5, Table S3, ESI). Jupyter notebooks for instrument control and machine learning are provided in the ESI. The experimental setup is shown in Figure S6.

## Results and Discussion

### Characterizing the Aptamer-Modified Electrode

Cyclic voltammetry (CV) was used to characterize the E-AB sensor, verify aptamer attachment to the electrode surface, and quantify aptamer density. We conducted all electrochemical measurements at room temperature in binding buffer (50 mM Tris (pH 7.5), 150 mM NaCl, 5 mM MgCl_2_) using the aptamer-modified gold working electrode, a saturated calomel reference electrode (SCE), and a platinum mesh counter electrode. All voltammetry in this work was performed in a potential range of −0.05 V to −0.4 V vs SCE to mitigate desorption of the SAM from the electrode surface.^43–47^ CV measurements were performed at a range of scan rates from 25 mV/s to 5 V/s. All voltammetry is plotted in Polarographic convention.

To confirm the successful fabrication of the sensor, CVs of the electrode were taken in binding buffer (Figure 2). The CV of the aptamer-modified electrode in Figure 2 shows a single surface-bound peak near the methylene blue reduction potential (−0.25 V vs SCE), characteristic of methylene blue-tagged aptamers,^14,48–50^ while the hexanethiol electrode exhibits only capacitive background. The CV of the aptamer in Figure 2 indicates a surface-bound species, having Gaussian potential–current profiles with no peak separation between the cathodic (forward) and anodic (reverse) peaks, and no diffusional tail. Additionally, scan rate study experiments (Figure S7) show a linear relationship between peak currents and scan rate, further supporting that our aptamer is successfully bound to the electrode surface.^51^ SWV measurements over 24 h show minimal signal loss, indicating a stable sensor (Figure S8). The surface coverage of the aptamer can be adjusted by changing the concentration of aptamer in the incubation solution and can be calculated as described in SI Note 1.^49,52^ Throughout this work we used 150 nM aptamer incubation, which resulted in an average surface coverage of 3.3 ± 0.2 × 10^12^ molecules/cm^2^.

**Figure 2:**
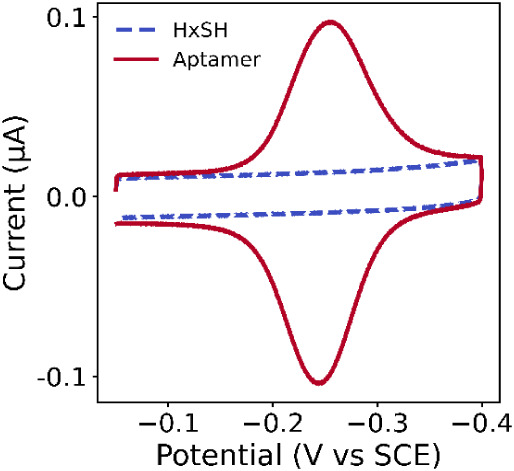
CV of the immobilized aptamer showing a surface-confined methylene blue peak (solid red trace) and a hexanethiol control (dashed blue trace) at 100 mV/s. Voltammetry is plotted in Polarographic convention.

### Automated Characterization of Sensor Response to Progesterone

The sensor response to progesterone was evaluated across a series of titration experiments (Figure 3) where we performed square wave voltammetry (SWV) measurements across a wide range of concentrations and frequencies to understand the on-target behavior of our sensor. Initially, the aptamer-modified electrode was equilibrated in binding buffer, and baseline SWV measurements were recorded. SWV measurements were performed using a potential step of 1 mV, an amplitude of 25 mV, and varying frequencies. Progesterone was then titrated into the solution in 1 μM increments, ranging from 0 to 30 μM, and the current response was recorded after each addition. From this titration data, the peak current values were extracted and plotted against progesterone concentration to generate a calibration curve. A representative titration experiment is shown in Figure 3, displaying the sensor’s response to increasing progesterone concentrations. This titration experiment was repeated 15 times to better understand the reproducibility of our aptamer modification. After ensuring that the titration data was reproducible, we performed a comprehensive combined titration and frequency mapping experiment. After each progesterone addition during the titration experiment described above, SWVs were taken at 40 frequencies ranging from 50 to 2000 Hz. We used the obtained frequency map to identify “on” and “off” frequencies that enhance or suppress the signal response, respectively. By recognizing the frequencies where the sensor response is maximized, we can pinpoint optimal interrogation conditions for the E-AB sensor.

**Figure 3:**
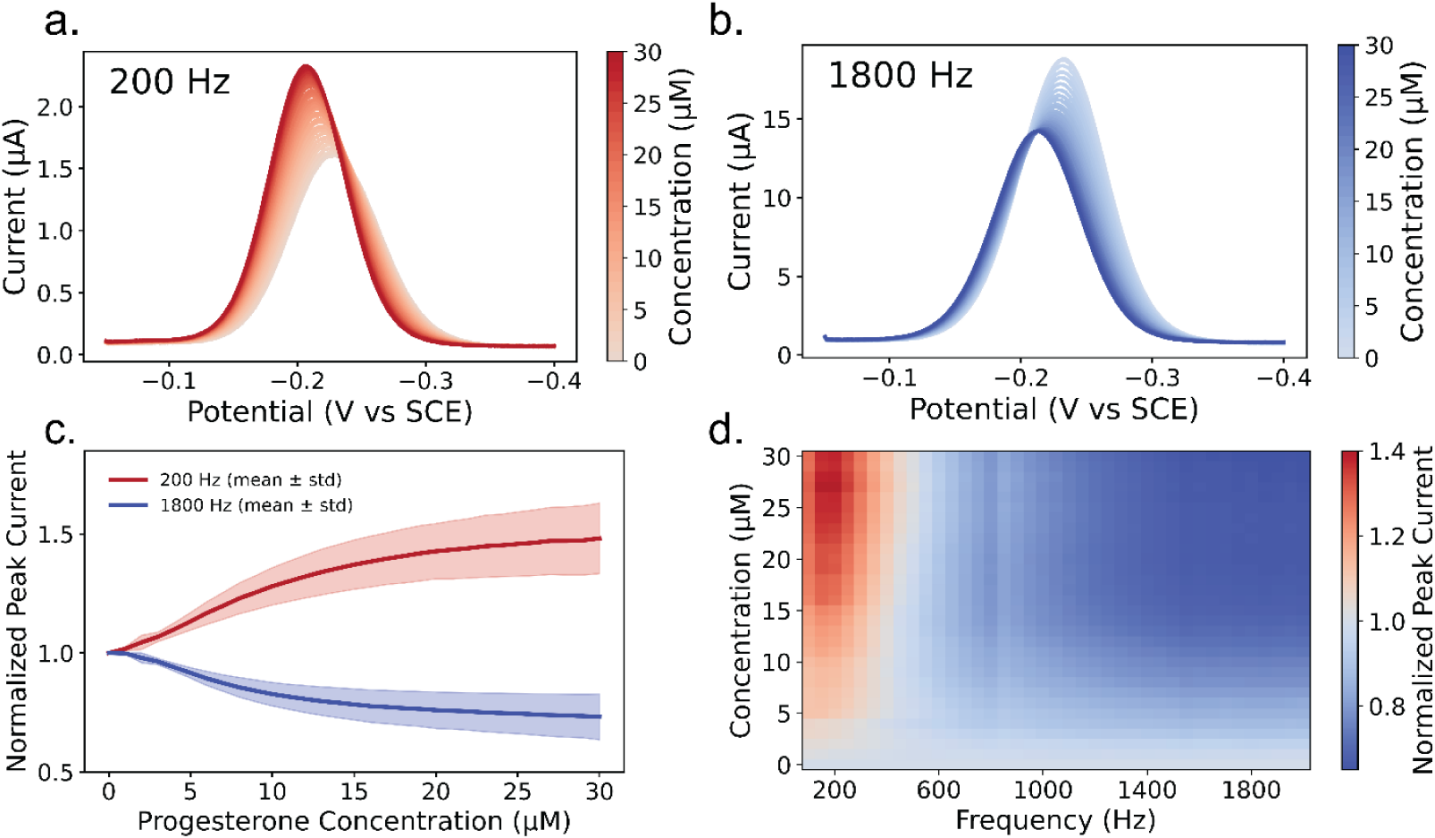
Aptamer sensor’s response to progesterone. Automated titration experiments conducted using square wave voltammetry (SWV) at (a) 200 Hz and (b) 1800 Hz with 1 μM titrations of progesterone from 0 to 30 μM. (c) A titration curve of normalized peak current plotted against progesterone concentration. Normalized peak current values were calculated by dividing the peak current from each titration SWV by the baseline peak current in the absence of progesterone. The shaded error bars represent 1 standard deviation from the mean (*N* = 15). (d) An automated titration and frequency mapping experiment where 40 frequencies between 50 and 2000 Hz were interrogated after each 1 μM titration of progesterone, across a concentration range of 0 to 30 μM. The normalized peak currents are plotted as a heat map, where dark red and dark blue regions can be used to identify “on” and “off” frequency regimes, respectively.

While these titration and frequency-mapping experiments can be performed manually, the process is labor-intensive and prone to variability. For example, manually performing the frequency-mapping experiment required over 8 h of continuous manual effort to collect 40 SWVs per each of the 31 concentration points (1,240 individual scans). Automation significantly streamlined this workflow, performing the same experiment in one-third of the time. Most notably, however, was the impact that automation had on experimental reproducibility. Variations in time between experimental operations, such as analyte addition, can have significant impacts on the experimental results. We achieved reproducible responses for 15 titrations performed over multiple electrodes and multiple days.

Automated titration experiments presented in Figure 3 demonstrate a clear signal response to progesterone, consistent with the structure-switching behavior of the aptamer. We see “signal-on” behavior at 200 Hz and “signal-off” behavior at 1800 Hz (Figure 3a and b, respectively), where the sensor response in the presence of progesterone is enhanced or suppressed, relative to the baseline signal taken in binding buffer. The different behavior at 200 and 1800 Hz arises from sampling the different electron transfer kinetics when the methylene blue redox tag is in either the bound or unbound state.^53^ This frequency-dependent response enables tuning of sensor behavior and provides an opportunity for self-referencing and calibration-free sensing.^14,18,49,53,54^ The concentration response curves indicate that the sensor follows the expected Langmuir-type binding behavior.^55^

Figure 3c shows the average normalized peak current as a function of progesterone concentration across 15 individual automated titration experiments. The normalized peak current at progesterone saturation reached 1.5 ± 0.1 in the signal-on regime, corresponding to a 50% increase in signal upon progesterone binding. Conversely, when probed in the signal-off regime the normalized peak current value plateaued at 0.73 ± 0.09, indicating a 27% suppression of signal. The reproducibility of these titration experiments helped us to confirm that our electrode modification procedure was reliable, and that the signal we were seeing was indeed due to aptamer-target binding. Additionally, we performed control experiments where we titrated in methanol to the buffer solution containing 5% methanol cosolvent, and no response was seen (Figure S9). Minimal drift was observed over repeated measurements, confirming that signal changes arose from progesterone addition rather than from the number of measurements performed or the addition of methanol cosolvent. We fit the average titration data to a Langmuir isotherm binding model to obtain the aptamer-target dissociation constant, *K*_d_.^49,56,57^ We obtained a *K*_d_ value of 22 ± 2 μM at 200 Hz and a *K*_d_ value of 17 ± 2 μM at 1800 Hz (Figure S10). We note that our *K*_d_ value is several orders of magnitude higher than the value obtained by fluorescence assay for the original aptamer sequence reported by Alhadrami and coworkers.^31^ However, this discrepancy between results from SWV and fluorescence assays is common when evaluating electrode-bound aptamers.^56^

To investigate the frequency-dependent behavior of the progesterone E-AB sensor, an automated frequency mapping experiment was conducted in which SWVs were collected across a broad range of frequencies (50–2000 Hz) and progesterone concentrations (0–30 μM). At each titration point, SWVs were collected at 40 different frequencies, generating a two-dimensional heat map of normalized peak current versus frequency and concentration, shown in Figure 3d. This heat map shows the two distinct signal regimes that are expected for E-AB sensors, as described above. As expected, we see that the signal increases with progesterone concentration at lower frequencies (dark red color in Figure 3d), while at high frequencies the signal decreases (dark blue color in Figure 3d). The signal increase peaks at 200 Hz, and the signal decrease appears to plateau after 1800 Hz. In between the “signal-on” and “signal-off” regimes, we see a transition region with a switching frequency at 600 Hz. These results demonstrate that square wave frequency acts as a powerful control parameter that can be tuned to optimize signal gain, and automation enables comprehensive screening for optimal conditions.

The titration and frequency mapping experiments in Figure 3 are a critical benchmark of sensor performance, helping us to understand the sensitivity of the sensor to its target molecule and compare it to other sensors through figures of merit such as linear dynamic range and the aptamer-target dissociation constant, *K*_d_. We highlight here the importance of reproducibility when performing these benchmark titration experiments, especially when evaluating sensors based on modified electrode surfaces. Surface modifications with self-assembled monolayers are prone to issues such as imperfect packing density, surface defects, and monolayer desorption, which can result in significant reproducibility issues that greatly impact overall sensor performance. Because of this, performing just one titration with a single modified electrode can be deceiving, with outlier samples potentially performing far better (or worse) than the average. To ensure that our characterization was comprehensive, we used automation to perform the exact same titration workflow across multiple electrodes over multiple days, ensuring that the average sensor performance was captured. This level of rigor when determining reproducibility would be excessive when performed manually; however, when automated, it dramatically reduces human error and variability in the experimental procedure. Importantly, we note that standardized automated characterization procedures could be developed, ensuring identical benchmarking across different laboratories.

### Automated Screening of Sensor Cross-Reactivity

Aptamers are affinity receptors and operate on the principle of molecular affinity rather than strict lock-and-key specificity like immunosensors. This means it is essential to evaluate their cross-reactivity toward a range of possible interferents to identify off-target responses, particularly when developing a sensor for continuous monitoring *in vivo*. To this end, we decided to perform an expansive study of our sensors’ cross-reactivity across a library of steroidal and nonsteroidal compounds. To evaluate the specificity of the aptamer sensor, we developed a comprehensive interferent scope selected from known blood metabolites in the Human Metabolome Database (HMDB).^40^ Dimensionality reduction of the chemical space via PCA and t-SNE was used to qualitatively guide interferent selection, ensuring that the selected library of interferents was not localized in chemical space (Figure S3). A final library of 40 possible interferents was selected (Figure 4a) including structurally related steroid hormones and precursors, small organic acids and fatty acids, amino acids and their derivatives, hydrophobic lipids and vitamins, redox cofactors, common pharmaceuticals and phenolics, sugars and energy metabolites, and other dietary and biologically relevant compounds.

**Figure 4:**
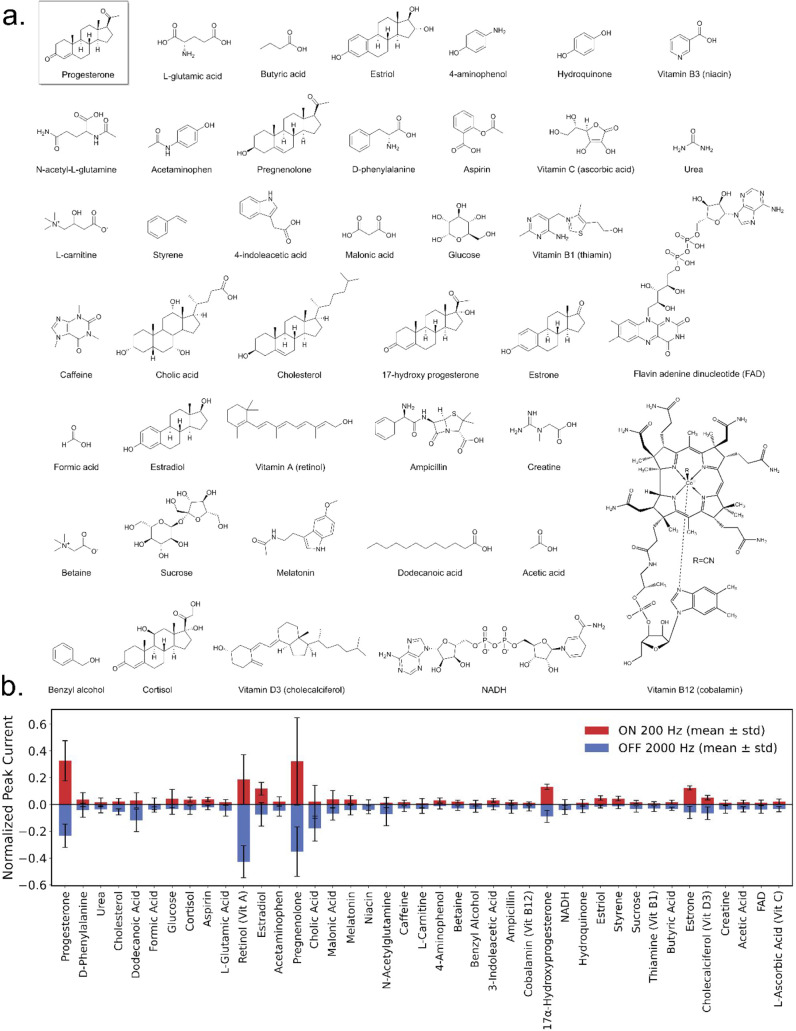
Measuring cross-reactivity of the progesterone sensor to a library of interferent molecules. (a) The 40 molecules in the interferent scope. (b) Sensor responses to interferents showing average normalized peak current and standard deviation across 9 trials at 200 Hz (red data) and 2000 Hz (blue data).

To screen such a large interferent scope, we established a high-throughput automated screening protocol, where the aptamer sensor was individually exposed to each compound in the interferent scope and SWVs were collected across a frequency range of 100–2000 Hz to identify optimal frequencies that minimized cross-reactivity. Our automated platform allowed us to perform identical screens with multiple different electrodes to ensure that batch-to-batch variability in electrode preparation was not affecting sensor selectivity. The automated screening protocol was as follows. Prior to substrate addition, the electrode was equilibrated in binding buffer and background SWVs were recorded to establish the baseline aptamer signal. A saturating concentration of each substrate (30 μM) was then titrated into the cell, followed by a 5 min binding period (optimized; see Figure S11). SWVs were collected postbinding, and the normalized peak current was calculated as the ratio of the background signal to the substrate-bound signal. Between each substrate, the system performed a rinse and debinding cycle to restore baseline conditions. To account for aptamer signal loss over time, each interferent’s response was compared to the background SWV recorded immediately prior to substrate addition. Additionally, progesterone was sampled at both the beginning and end of each experimental sequence to monitor temporal changes in aptamer performance. For each electrode, we performed this experimental sequence three times with a randomized order of interferents to average out any possible memory effects. In total, the response for each interferent was measured nine times. As a control, CVs of a hexanethiol-modified electrode were taken in the absence and presence of each interferent to confirm no electrochemical activity of interferents in the potential window sampled (Figure S12).

Figure 4a shows the 40 molecules in our interferent scope and Figure 4b shows the sensor’s response to each interferent molecule at 200 Hz (red data) and 2000 Hz (blue data), plotted as normalized peak current. The “signal-off” frequency was selected based on frequency mapping experiments, which showed that the signal plateaued above ∼1800 Hz. Accordingly, either 1800 or 2000 Hz was used as the “signal-off” frequency, as negligible difference in normalized signal was observed between these values. The normalized peak current values in Figure 4b are centered around zero, with values above zero indicating a “signal-on” response, and values below zero indicating a “signal-off” response. Looking at the data in Figure 4b we can immediately see that our sensor responded to multiple interferent molecules. We observed measurable electrochemical responses not only to progesterone but also to structurally similar steroidal compounds and, surprisingly, some structurally unrelated nonsteroidal compounds. However, most interferent molecules showed negligible change in “signal-on” and “signal-off” responses after substrate addition.

Many of the steroidal molecules that are structurally similar to progesterone yielded elevated current responses, including estradiol, pregnenolone, 17α-hydroxyprogesterone, estrone, and cholic acid. Such cross-reactivity of our sensor to steroid molecules is unsurprising, and a common problem in affinity-based steroid sensors.^21^ Several molecules, such as pregnenolone, exhibit relatively high variability in signal, which we attribute primarily to day-to-day differences in sensor fabrication. Because the automated workflow provides consistent and reproducible screening conditions, this variability reflects fluctuations in sensor chemistry rather than the measurement process. This effect is particularly evident for pregnenolone, where one experimental day yielded consistently lower responses. Automating electrode fabrication could reduce this source of variability and represents an important direction for improving reproducibility in future studies. Besides steroidal cross-reactivity, we observed pronounced responses from nonsteroidal molecules such as vitamin A (retinol) and dodecanoic acid. Structurally, both retinol and dodecanoic acid present extended hydrophobic, aliphatic surfaces, which could promote nonpolar contacts with the aptamer or electrode interface. Aptamers, composed of nucleic acids with a highly polar sugar–phosphate backbone, struggle to generate the deep, sterically well-defined hydrophobic cavities characteristic of other biorecognition elements such as enzymes.^58–63^ Because of this, it is likely that long aliphatic molecules can interact promiscuously via nonspecific hydrophobic and van der Waals contacts. However, we note that the hexanethiol backfill layer in our electrode scaffold presents a dense array of terminal methyl groups that form a hydrophobic surface layer, which could interact with hydrophobic interferents.^64,65^ In fact, monolayer end group chemistries as well as alkyl chain length and thiol anchoring group and have been shown to directly affect the biocompatibility, operational stability, and signal gain of E-AB sensors.^66–69^ The responses that we see are likely a convolution of nonspecific hydrophobic interactions with the aptamer and with the hydrophobic backfill monolayer. Future work must be done to investigate this possibility and disentangle the root causes of this behavior. Although hexanethiol produces a hydrophobic surface that may contribute to nonspecific interactions, it was selected based on prior work demonstrating that densely packed alkanethiol monolayers improve E-AB sensor performance through reduced capacitive currents, increased Faradaic signal, and enhanced monolayer stability in aqueous and biological environments compared to more commonly used polar backfill monolayers such as mercaptohexanol.^43^ Notably, preliminary studies indicate that hydrophilic monolayers completely attenuate sensor response, a phenomenon we are actively investigating.

During our interferent study we collected SWVs at a range of frequencies for each interferent, aiming to provide insight into the dynamics of off-target binding. The frequency map in Figure 5a shows normalized peak current across a wide SWV frequency range for each interferent (labeled frequency map shown in Figure S13). The frequency map presents the complete spectral response across the entire interferent scope and visually highlights regions of maximum and minimum signal (red and blue, respectively). Notably, many interferents show different optimal “signal-on” and “signal-off” frequencies than those of progesterone. This provides us with a handle to tune our sensor’s selectivity by judiciously choosing the interrogation frequencies. To this end, we developed an equation (eq 1) to describe the selectivity of our sensor against the screened interferents:

1 $$Selectivity=\frac{\left|S_{target}\right|}{\frac{1}{N}\overset{N}{\underset{i=0}{\sum }}\left|S_{i}\right|}$$

**Figure 5:**
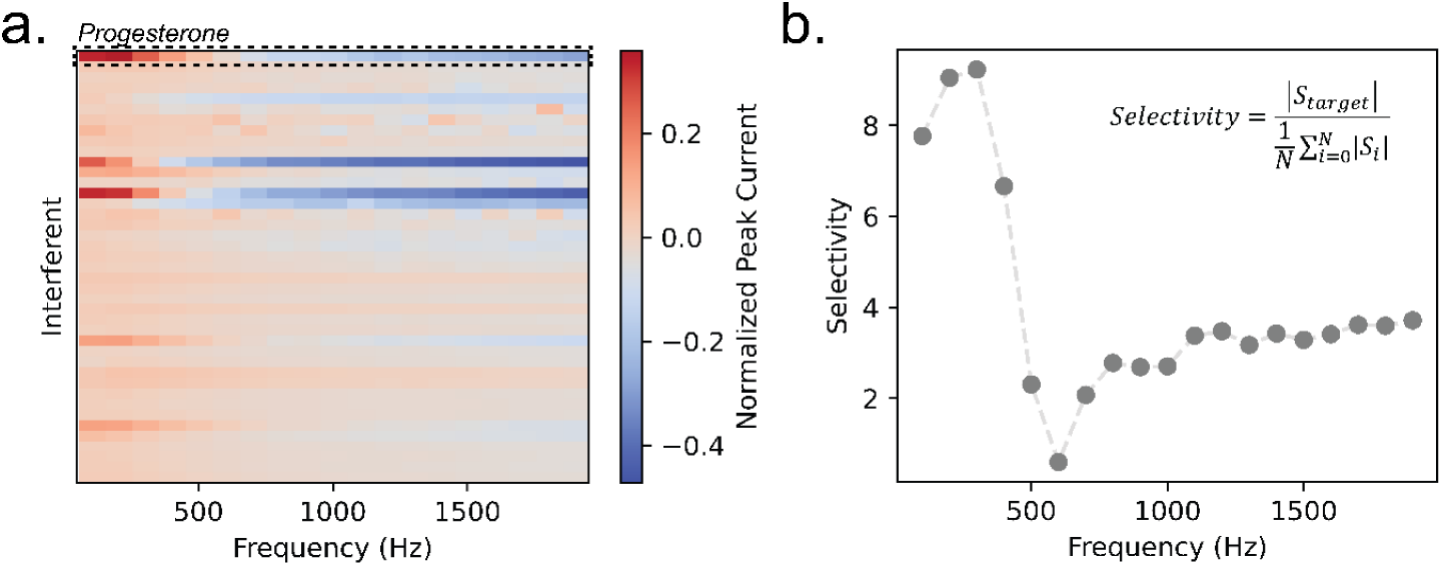
(a) Heat map showing the average normalized peak current for each interferent across 20 frequencies ranging from 100–2000 Hz. High “signal-on” responses are indicated by a higher normalized peak current value and are plotted in red. High“signal-off” responses are indicated by a more negative normalized peak current value and are plotted in blue. (b) Selectivity calculated using eq 1 (shown in the inset) plotted as a function of SWV frequency.

The selectivity of our sensor was calculated using eq 1 for each frequency in our experiment. Figure 5b shows the calculated selectivity plotted as a function of frequency, with a peak at 300 Hz, identifying the frequency where progesterone selectivity was maximized. We note that this frequency would likely change depending on the molecules included in the interferent scope.

In total, during the interferent screen, 15,840 SWVs were collected over 6 days of continuous automated experimental runtime. The throughput and reproducibility afforded by automation were essential to the feasibility of this study. Without automated solution handling, substrate delivery, and data acquisition, the experimental scope would have required an estimated three months of continuous manual labor. We again note that our automated screening protocol enabled precise control over experimental conditions, including substrate concentration, binding time, and rinsing protocols, ensuring a consistent treatment across all trials. This level of reproducibility is especially critical in interferent screening, where subtle differences in aptamer response can now be attributed to molecular specificity rather than experimental inconsistencies.

### Machine Learning to Understand Sensor Cross-Reactivity

When we initially began our efforts to understand cross-reactivity, we hypothesized that a molecule’s structural similarity to progesterone would be the key descriptor of off-target response. However, the unexpected response from nonsteroidal interferents such as retinol and dodecanoic acid contradicted our hypothesis. To fully understand this unexpected result, we turned to an interpretable machine learning approach to identify physically meaningful descriptors that could explain the large signal response from nonsteroidal molecules. Our automated screening of sensor cross-reactivity provided us with an appropriate amount of high-quality data to train meaningful machine learning models. We developed a multioutput machine learning model that related computationally derived descriptors to the responses shown in Figure 4b.

To develop our model, we first selected a set of physically informative descriptors. We calculated RDKIT descriptors for each compound in our interfering library.^70^ From the complete set of RDKIT descriptors, we selected ones that were intuitive and physically meaningful, manually removing highly correlated and physically unintuitive features. We further performed feature pruning through successive modeling and feature permutation rankings. Finally, we arrived at 7 features that we felt were meaningful and intuitive: Tanimoto similarity to progesterone (Similarity), computational water-octanol partition coefficient (MolLogP),^71^ van der Waals volume (Volume), number of aliphatic carbocycles (Aliphatic Carbocycles), weighted number of ketones (Ketone Descriptor), fraction of carbons that are sp^3^ (FractionCSP3), and topological polar surface area (TPSA). Figure 6a shows abstract representations of each descriptor.

**Figure 6:**
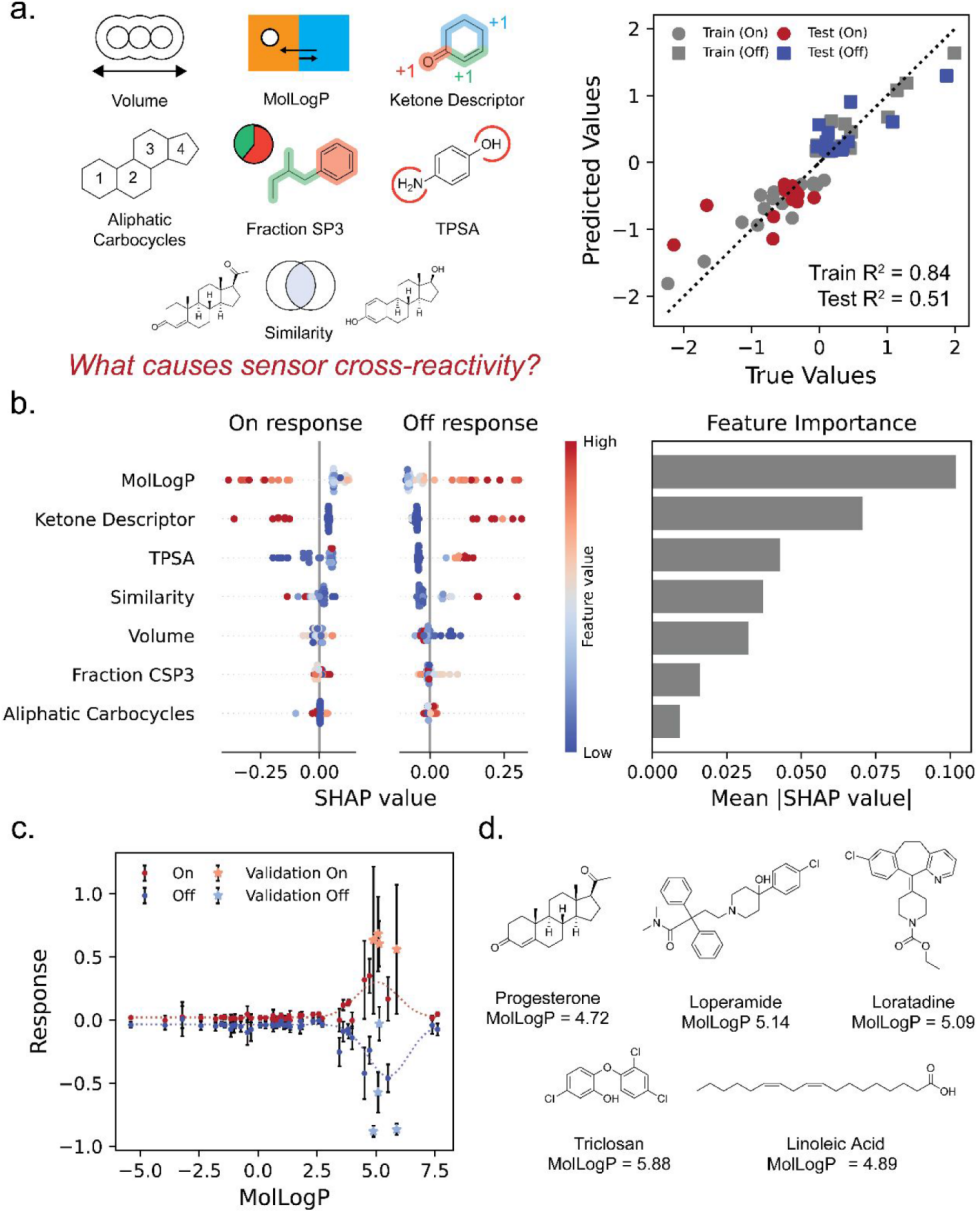
Using machine learning to identify features that impact cross-reactivity. (a) Potentially impactful molecular features (left) were used to train a multioutput random forest (RF) model with training *R* ^2^ of 0.84 and a test *R* ^2^ of 0.51 (right). (b) SHAP analysis showing impact of the features on both RF model outputs (on and off response) and the average absolute impact on the model results. Of all the features, MolLogP (computationally computed LogP) showed the highest averaged absolute SHAP value. (c) A plot of sensor response against MolLogP for the interferent scope, and (d) the external validation set of compounds with MolLogP values close to 5.

We used the descriptors in Figure 6a as our model inputs and the average sensor response at 200 and 2000 Hz as our model outputs. A 60:40 train-test split was done, and model architecture and hyperparameter screening were carried out on the training data using leave-one-out cross-validation scored on negative mean squared error (MSE). The modeling architectures screened included Kernel Ridge Regression, Support Vector Regression, k-Nearest Neighbors Regression, and Random Forest Regression (Figure S14). The best model was a Random Forest regressor (100 estimators, max depth of 3) with a train *R* ^2^ of 0.84 and a test *R* ^2^ of 0.51, as shown in Figure 6a. Although the predictive performance is modest, the purpose of the model is interpretability rather than high-accuracy prediction, enabling the extraction of physically meaningful descriptors. While the specific model is not generalizable to other aptamer systems, the use of interpretable machine learning techniques provides a means to identify key molecular features for the purpose of hypothesis generation, which can then be validated experimentally. SHAP analysis was carried out on our model to understand the impact that the chosen descriptors had on the modeled sensor response.^72,73^ Figure 6b shows the directional impact of each feature on the modeled “signal-on” and “signal-off” response, as well as the average absolute impact on the model. Overall, the most impactful descriptor on sensor response was MolLogP, the computationally derived octanol–water partition coefficient.^71^ Figure 6c shows the measured sensor response as a function of MolLogP, with distinct peaks for both the “signal-on” and “signal-off” responses centering around MolLogP values of ∼5. Based on the clear presence of this peak, we hypothesized that the aptamer was not strictly sensitive to progesterone, but instead it has an apparent affinity for any molecule of similar hydrophobicity. To test this, we performed a validation screen with four extra interferents (Figure 6d) that had MolLogP values close to 5 but were very structurally different from progesterone. The responses (Figure 6c, Figure S15) for these four interferents showed the highest response of all interferents screened. This clearly indicated that hydrophobicity was the key molecular feature that determined sensor cross-reactivity, much more so than structural or functional motifs (Figure S16). The observed dependence on hydrophobicity is reasonable, as steroid molecules have limited polar functional groups and must primarily engage in nonspecific hydrophobic affinity binding with the aptamer.^21^

The observed cross-reactivity toward retinol and all four validation molecules likely reflects broader interactions with lipophilic compounds of similar logP values present in biological fluids. Biological fluids contain a wide variety of structurally related lipophilic molecules (e.g., steroids, lipids, fat-soluble vitamins) that compete for binding under physiological conditions leading to nonspecific interactions or competitive binding.^3^ Consequently, evaluation in complex matrices and incorporation of selectivity-enhancing strategies will be critical for translation into practical diagnostic devices.^2^ To this end, surface functionalization to reduce nonspecific adsorption,^47,68,74^ calibration in relevant biological matrices (serum, plasma, whole blood),^3,75^ and use of differential measurements^57^ are three mitigation strategies employed to address selectivity issues in complex media. Additionally, the sensor’s ability to detect any molecule with MolLogP values close to that of typical steroidal hormones presents a great opportunity to expand the application of this technology. There are many molecules that have MolLogP values of ∼5 that are known endocrine-disrupting chemicals (EDCs) that are hazardous to human health. EDCs (for example, BPA, phthalates, dioxins, and PFAS) compete with endogenous hormones for receptor binding sites, perturbing hormonal homeostasis and causing developmental and reproductive abnormalities.^76^ We posit that the selectivity of our sensor to molecules with a very specific range of relevant MolLogP values could be advantageous when detecting for possible environmental exposure to EDCs, enabling timely monitoring and intervention to reduce reproductive, metabolic, and hormone-dependent disease risks from chronic EDC exposure.^77–79^

## Conclusions

In this work, we studied progesterone sensing as a test bed to evaluate aptamer-based systems that have known complexities in selectively discriminating between steroid molecules. We developed a label-free E-AB sensor and used an automated electrochemistry platform based on eLab and Hard Potato to comprehensively characterize our sensor’s response to progesterone through titration and frequency mapping experiments. Automating our experiments greatly improved the reproducibility of our characterization workflow and increased experimental throughput drastically. During this work, we collected over 20,000 SWVs across many different frequencies, concentrations, analytes, and electrodes, demonstrating the high-throughput capability of our platform. We used our automated platform to screen an interferent scope of 40 molecules, identifying several molecules that gave a prominent response. We turned to machine learning and chemoinformatics techniques to understand the effects that molecular descriptors such as structural similarity and hydrophobicity have on aptamer cross-reactivity. From the data collected in the interferent scope screen, we developed a machine learning model that identified hydrophobicity as a key descriptor in off-target sensor response, validating this by testing a subset of molecules unseen by the machine learning model. Our results provide a quantitative framework for diagnosing why steroid-binding aptamers fail in complex environments, and how next-generation sensors can be rationally improved. Throughout this project we identified two key takeaways that will be useful to other researchers. First, automation significantly improves the reproducibility of characterization and screening protocols. Using an automated electrochemistry platform enabled systematic exploration of high-dimensional sensing parameter spaces (frequency, concentration, analyte identity, and electrode variability) that are otherwise experimentally inaccessible, revealing new chemical insights about aptamer-based sensors. We hope to see this adopted by the community, so that standardized characterization and screening protocols can be shared between laboratories, promoting transparency and reproducibility in the sensing community.^80^ Second, we hope to encourage the sensing community to increase the diversity of their interferent scopes, taking note from similar movements in organic synthesis.^38,81^ Many works in the sensing field screen structurally similar molecules to the analyte of interest, but this does not capture all possible off-target interactions, which can have implications on improving aptamer selection processes.^82^ By fully understanding the response to diverse interferents, we can better inform and design counter-selection processes that will improve aptamer selectivity and sensitivity.^49^

Overall, we believe that our work addresses an underexplored intersection of electrochemical sensing, laboratory automation, and machine learning. We believe that the field of electrochemical sensors will see accelerated discovery and improved reproducibility upon the integration of high-throughput and data-driven methodologies into traditional sensing research. Our findings regarding the cross-reactivity of our aptamer will be of interest to researchers designing novel oligonucleotide sequences. We hope that this work will motivate other researchers to incorporate data-driven methodologies into their labs.

## Supplementary Material





## Notes

ESI: raw data for titration and interferent experiments, code used to control the automated platform (ZIP)
Additional details and figures on aptamer structure, the automated platform, voltammetry, frequency mapping, interferent response, and machine learning models; Further overview of the automated eLab platform is shown in this YouTube video: https://youtu.be/0ycrwRe7sQs?si=QKo2J0GSUrPmgF1U (PDF)
