# Supplementary material for: Data-Driven Electrochemistry Reveals the Impact of Hydrophobicity on Aptamer Cross-Reactivity
Source: ACS Meas Sci Au. 2026 May 20;6(3):752–64. doi: 10.1021/acsmeasuresciau.6c00040 (PMC13281204; doi:10.1021/acsmeasuresciau.6c00040)
Supplement: Supplementary file 2 [file tg6c00040_si_002.pdf]

Data-Driven Electrochemistry Reveals the Impact of Hydrophobicity on Aptamer Cross Reactivity

Emily Carroll[a], Michael A. Pence[b,*], Elizabeth Winterholler[c], Taylor D. Sparks[c], Shelley D. Minteer[b,*]

[a]Department of Chemistry, University of Utah, Salt Lake City, Utah 84112, United States
[b]Kummer Institute Center for Resource Sustainability, Missouri University of Science and Technology, Rolla, MO 65409
[c]Department of Materials Science & Engineering, University of Utah, Salt Lake City, Utah 84112, United States

[*]Corresponding Author
Michael A. Pence- mike.pence@utah.edu
Shelley D. Minteer – shelley.minteer@mst.edu

Table of Contents

		Page
Figure S1	2D aptamer structure from mfold	3
Table S1	Solvent preparation conditions for interferent study	4
Figure S2	Structure of the modified DNA probe	5
Figure S3	Interferent downselection from HMDB	6
Figure S4	CVs of electrochemical cleaning of the electrodes	7
Table S2	Solution conditions used to prepare varying concentrations of aptamer	7
Figure S5	Solenoid driver device schematic	8
Table S3	Bill of materials for the gas switching device	8
Figure S6	Photos of experimental setup	9
Figure S7	Voltammetric characterization (CV scan rate study and peak current analysis) of immobilized aptamer	10
Figure S8	SWV stability study of aptamer-modified electrode	10
SI Note 1	Calculating the surface coverage of the aptamer-modified electrode	11
Figure S9	Methanol titration control	12
Figure S10	Fitting of titration data and calculation of K_d	13
Figure S11	Optimized binding study of progesterone	14
Figure S12	Background CVs of interferent molecules	15
Figure S13	Labeled frequency map of 40 interferent molecules plus progesterone	16
Figure S14	Comparison of different machine learning models tested in this work	17
Figure S15	Response of aptamer sensor to MolLogP validation molecules	17
Figure S16	Sensor response as a function of the various descriptors	18

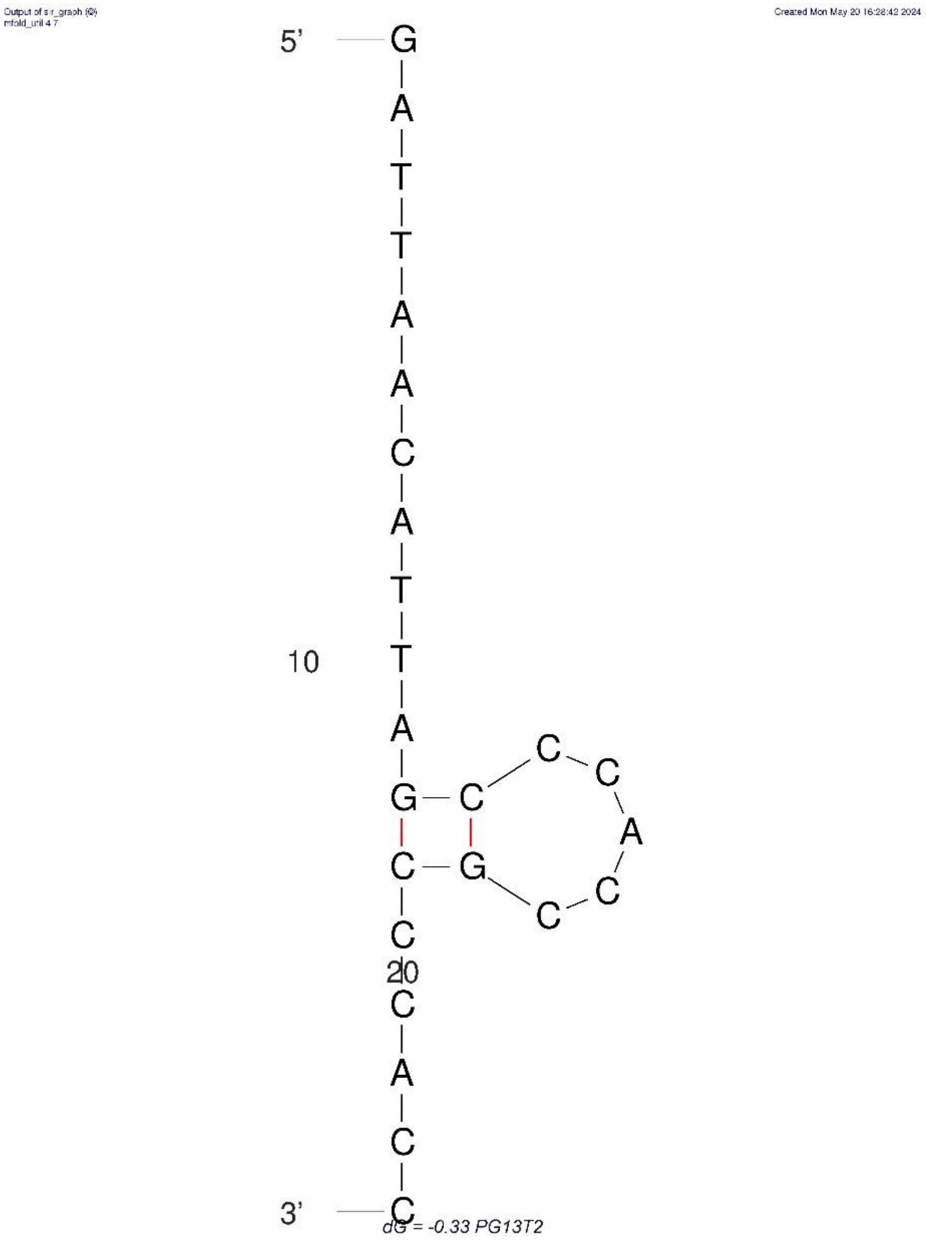

Figure S1. Aptamer secondary structure predicted from UNAfold web server.[1]

Table S1. Solvent preparation conditions for interferent study.

Interferent molecule name	Preparation conditions
Progesterone	Methanol
D-phenylalanine	Water
Urea	Water
Cholesterol	Methanol
Dodecanoic acid	Methanol
Formic acid	Water
Glucose	Water
Cortisol	Methanol
Aspirin	Methanol
L-glutamic acid	Water
Retinol (Vit A)	Methanol
Estradiol	Methanol
Acetaminophen	Water
Pregnenolone	Methanol
Cholic acid	Methanol
Malonic acid	Water
Melatonin	Methanol
Niacin (Vit B3)	Water
N-acetylglutamine	Water
Caffeine	Water
L-carnitine	Water
4-aminophenol	Methanol
Betaine	Water
Benzyl alcohol	Water
3-indoleacetic acid	Methanol
Ampicillin	Water
Cobalamin (Vit B12)	Water
17α-hydroxyprogesterone	Methanol
NADH	Water
Hydroquinone	Water
Estriol	Methanol
Styrene	Methanol
Sucrose	Water
Thiamine (Vit B1)	Water
Butyric acid	Water
Estrone	Methanol
Cholecalciferol (Vit D3)	Methanol
Creatine	Water
Acetic acid	Water
FAD	Water
L-ascorbic acid (Vit C)	Water
Triclosan	Methanol
Linoleic acid	Methanol
Loratadine	Methanol
Loperamide	Methanol

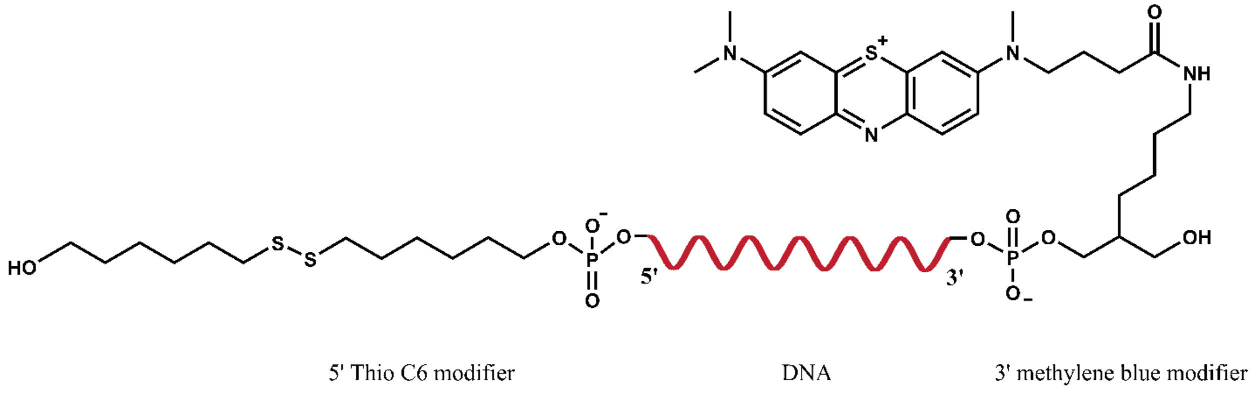

Figure S2. Structure of the thiolated, MB-modified DNA probe employed in this work for the fabrication of E-AB sensors (DNA component shown in red).

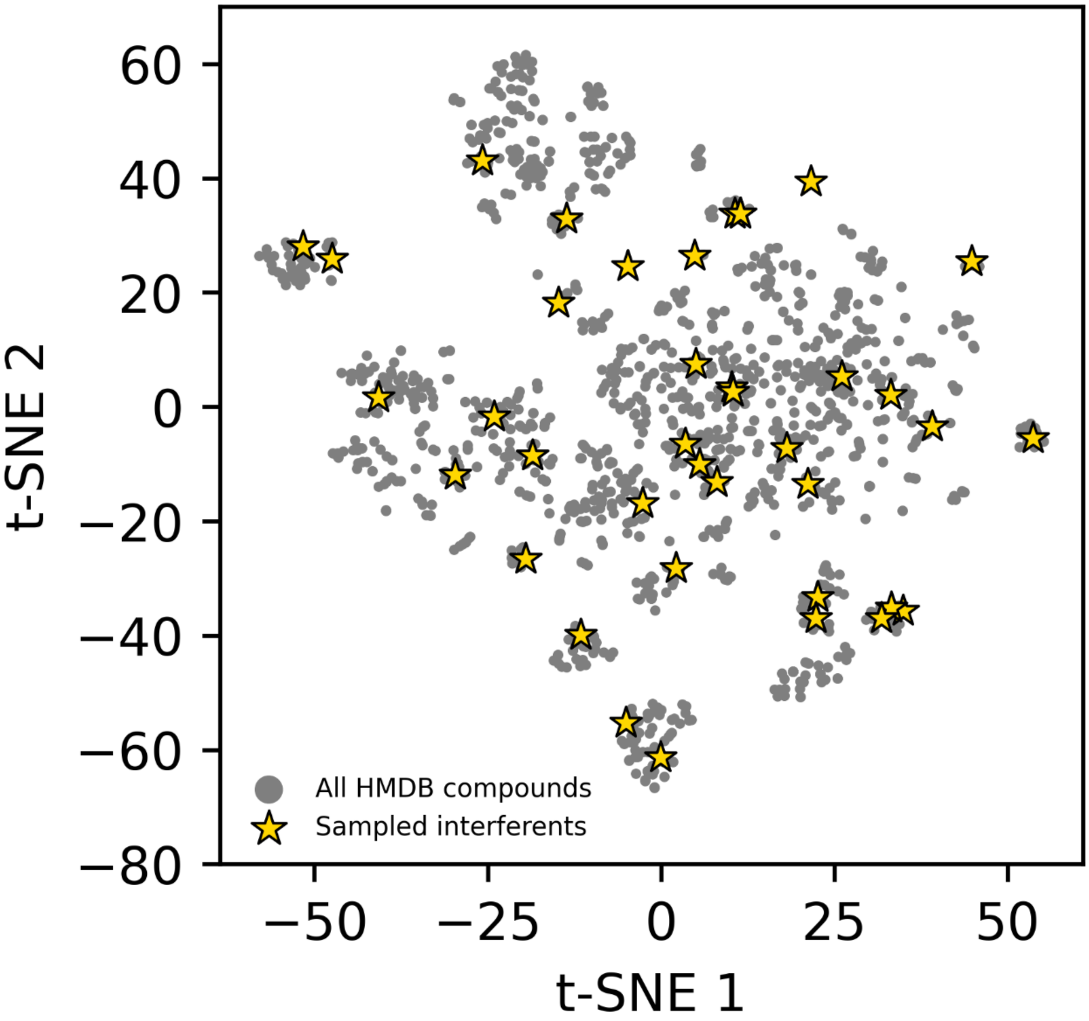

Figure S3. Map of chemical space generated by performing dimensionality on Morgan fingerprints of the HMDB, with interferents sampled in this work shown by starred points.

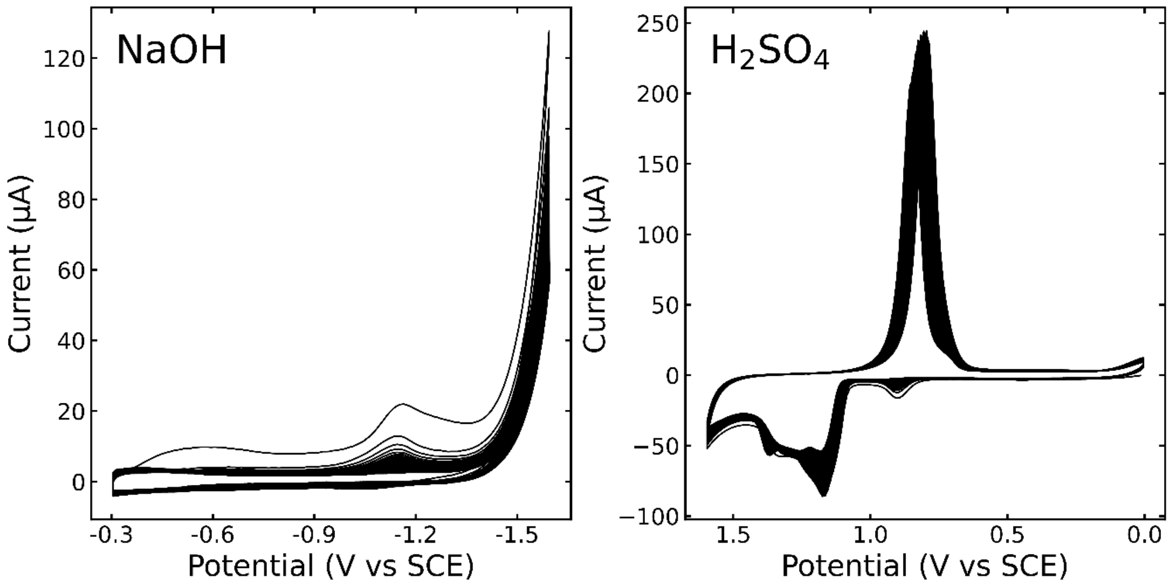

Figure S4. Electrochemical cleaning of the electrodes before immobilization of aptamer. The electrode is cycled in 0.5 M NaOH for 200 cycles over the potential range -0.3 V to -1.6 V vs. SCE (scan rate, 0.5 V/s; sample interval, 0.01 V). Next, the electrode is cycled in 0.5 M H_2SO_4 for 200 cycles over the potential range 0 V to 1.6 V vs. SCE (scan rate, 0.5 V/s; sample interval, 0.01 V).

Table S2: Solution preparation conditions used when preparing the aptamer-modified electrode at different incubating concentrations of aptamer.

Aptamer concentration	Volume aptamer (from 50 μM stock)	Volume TCEP (from 5 mM stock)	Volume buffer	Total incubating volume
100 nM	1 μL	1.5 μL	497 μL	500 μL
150 nM	1 μL	1.5 μL	330 μL	333 μL
200 nM	1 μL	1.5 μL	247 μL	250 μL
500 nM	2 μL	3 μL	195 μL	200 μL

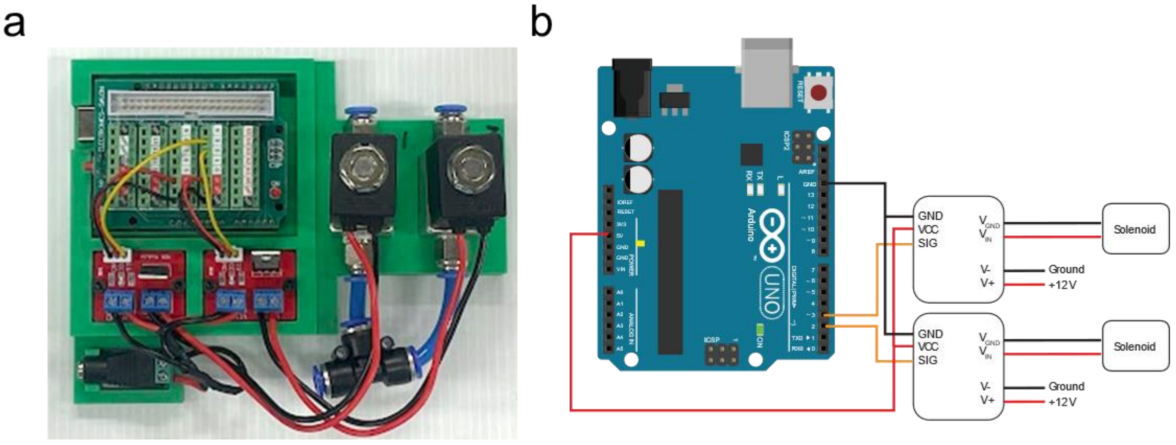

Figure S5. Solenoid driver device for controlling gas flow. (a) Photograph of the device and (b) schematic of the device.

Table S3. Bill of materials for the gas switching device.

**Item**	**Quantity**	**Price**	**Link**
Arduino	1	$13.99	https://www.amazon.com/ELEGOO-Controller-ATmega328P-Compatible-Arduino/dp/B0B6VV7MS7
Arduino Terminal	1	$18.99	https://www.amazon.com/Electronics-Salon-Arduino-Terminal-Breakout-Module/dp/B07HF2DD7T/
Valve	2	$8.49	https://www.amazon.com/Beduan-Normally-Closed-Electric-Solenoid/dp/B07N2DZ5FP
Valve driver	1	$6.99	https://www.amazon.com/HiLetgo-IRF520-MOSFET-Arduino-Raspberry/dp/B01I1J14MO
Power Supply	1	$6.99	https://www.amazon.com/Supply-Universal-Adapter-AC120V-Transformer/dp/B0C22Z8DPL
**Total**	$63.94		

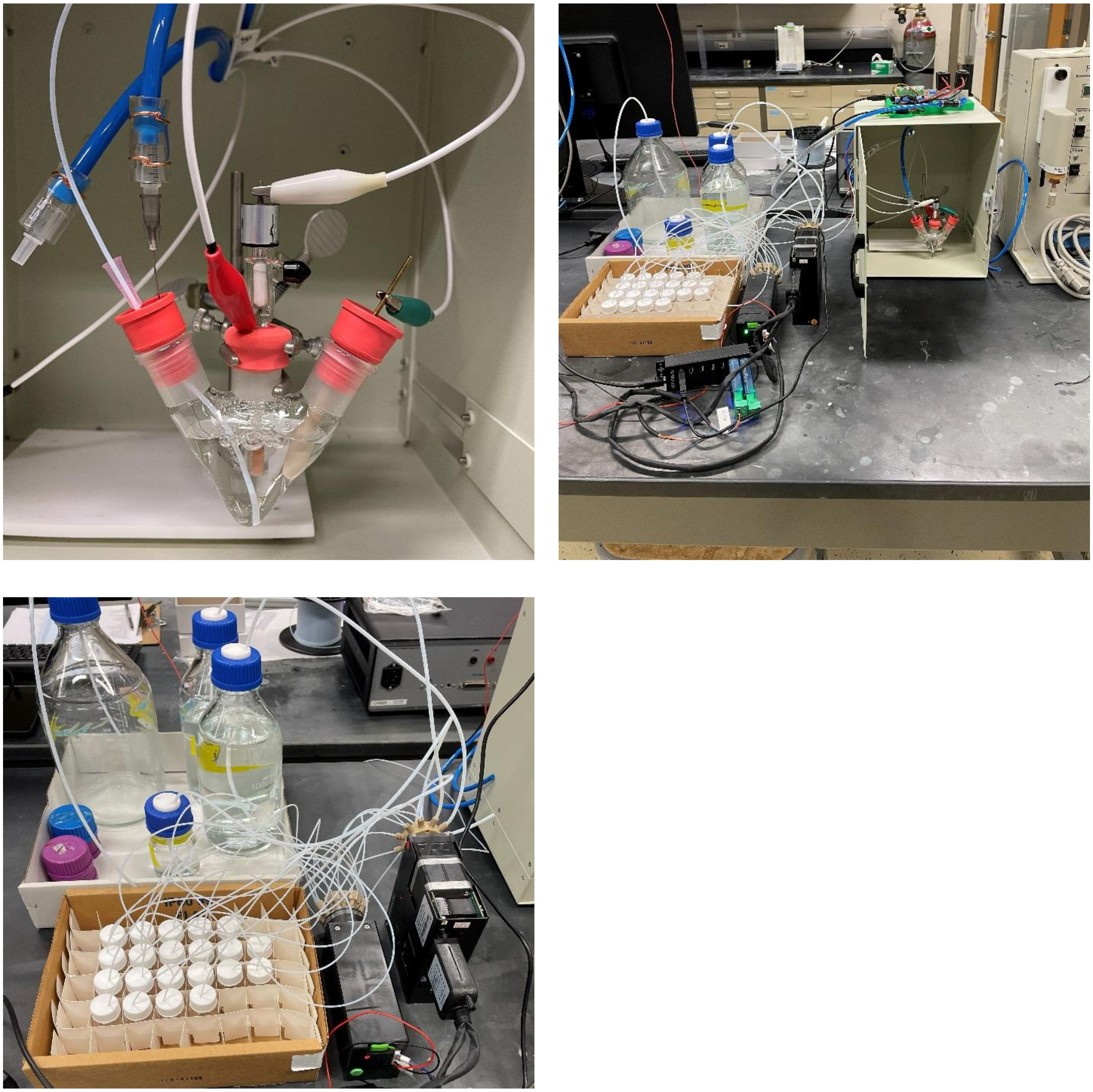

Figure S6. Photos of experimental setup. **Top left:** electrochemical cell showing modified gold working electrode, SCE reference electrode, platinum mesh counter electrode, argon needle, and robot input tube. **Top right:** titration robot setup including buffer bottles, automatic gas sparging, and interferent scintillation vials connected to syringe pump. **Bottom left:** closer image of interferent scope vials and buffer stock connected to syringe pump.

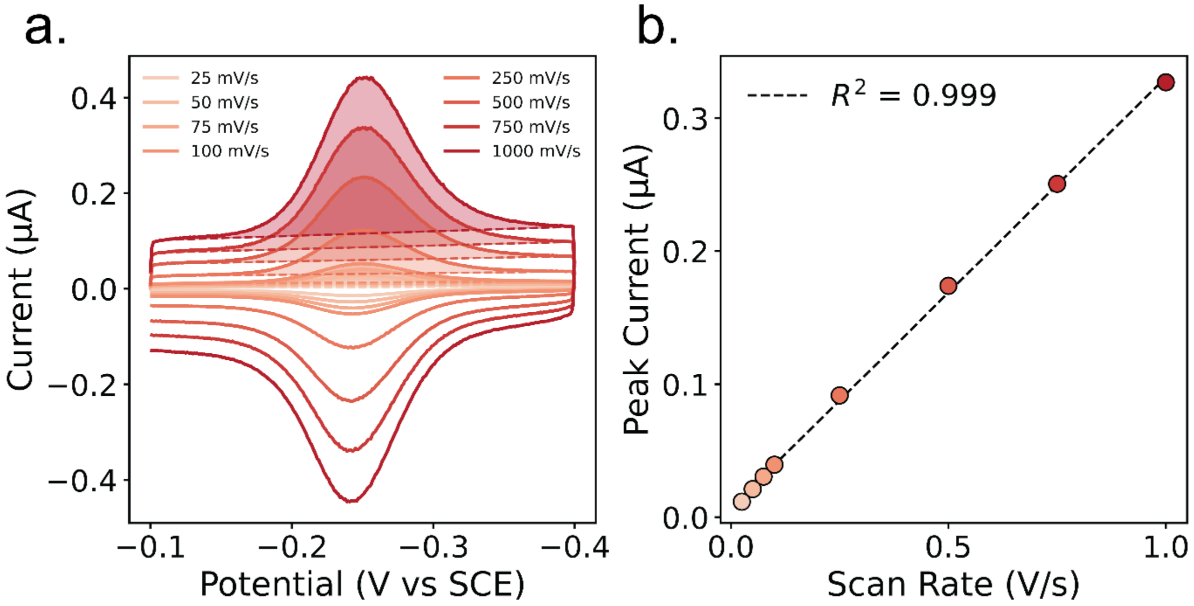

Figure S7. Voltammetric characterization of immobilized aptamer. (a) Scan rate study showing scan rates ranging from 25 mV/s to 1000 mV/s, highlighting the baseline subtraction (dotted line) and shaded area corresponding to the charge passed used for surface coverage calculations. (b) Correlation plot between peak current and scan rate showing a linear dependence for the surface confined aptamer. Voltammetry is plotted in Polarographic convention.

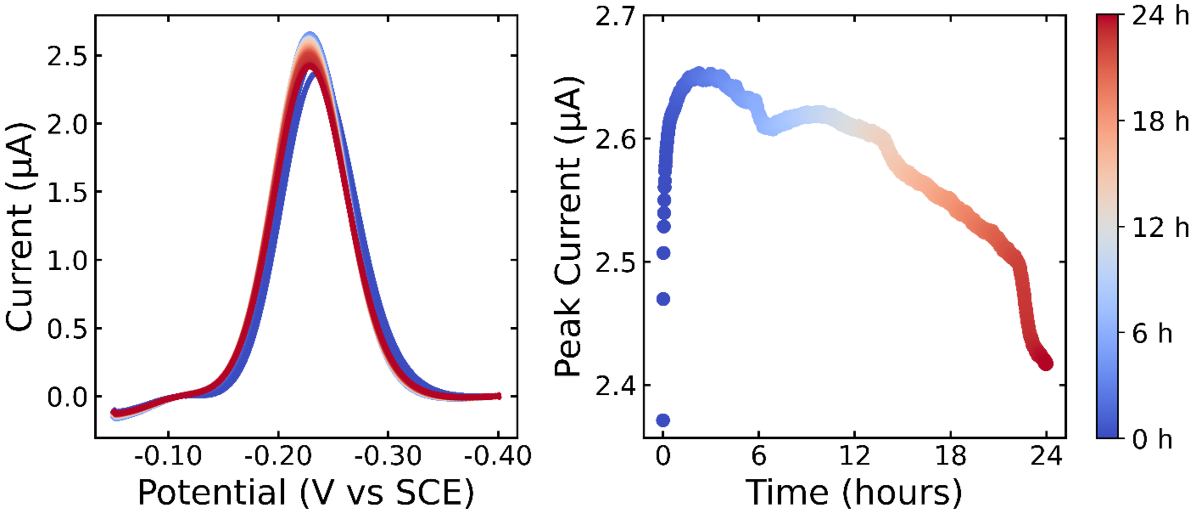

Figure S8. SWV stability study showing minimal (1.93%) change in peak current over 24 hours. **Left:** SWV data, baseline subtracted. **Right:** peak current vs time tracking how the current changes every minute over 24 hours.

SI Note 1 – Calculating the surface coverage of the aptamer

Typically, electrodes are incubated in alkylthiol solution of 1 mM. After self-assembly, the final typical adsorbate coverage of a strictly alkylthiol monolayer is 10^{-10} to 10^{-9} mol/cm^2 (6x10^{13} to 6x10^{14} molecules/cm^2).[2] When forming a mixed monolayer of alkylthiol and aptamer, the surface coverage of the aptamer can be calculated by measuring the amount of charge passed from the reduction of methylene blue, assuming one methylene blue molecule per oligo strand. Faraday's law of electrolysis (eq. S1) is used to find the number of moles reacted given the total charge passed (Q), the number of electrons transferred (n=2 for methylene blue), and Faraday's constant (approximately 96,500 C/mol). The surface coverage of the aptamer (Γ^*) can be calculated by dividing the number of moles by the area of the electrode (eqs. S2 & S3).

$$Q = nFN \qquad \textit{Equation S1}$$

$$Q = nFA\Gamma^* \qquad \textit{Equation S2}$$

$$\Gamma^* = \frac{Q}{nFA} \qquad \textit{Equation S3}$$

Due to the bulky nature of aptamers, the typical aptamer surface coverage is 10^{11} to 10^{12} molecules/cm^2, several orders of magnitude less than a strictly alkylthiol monolayer.[3-5] The optimal packing density varies depending on the tertiary structure and folding dynamics of the aptamer, as well as the size of the target molecule or protein. At high surface densities, steric hindrance between adjacent aptamers can impede proper folding and reduce target accessibility, which in turn may alter the apparent binding affinity (K_d) of the sensor.[5] Conversely, if the packing density is too low, the limited number of surface-bound aptamers may generate insufficient faradaic current to distinguish signal from background noise.[6] Thus, achieving an appropriate balance in aptamer surface coverage is critical for maximizing sensor performance. Throughout this work we used 150 nM aptamer incubation, which resulted in an average surface coverage of $3.3 \pm 0.2 \times 10^{12}\ \mathrm{molecules/cm^2}$ or $5.5 \pm 0.4 \times 10^{-12}\ \mathrm{mol/cm^2}$.

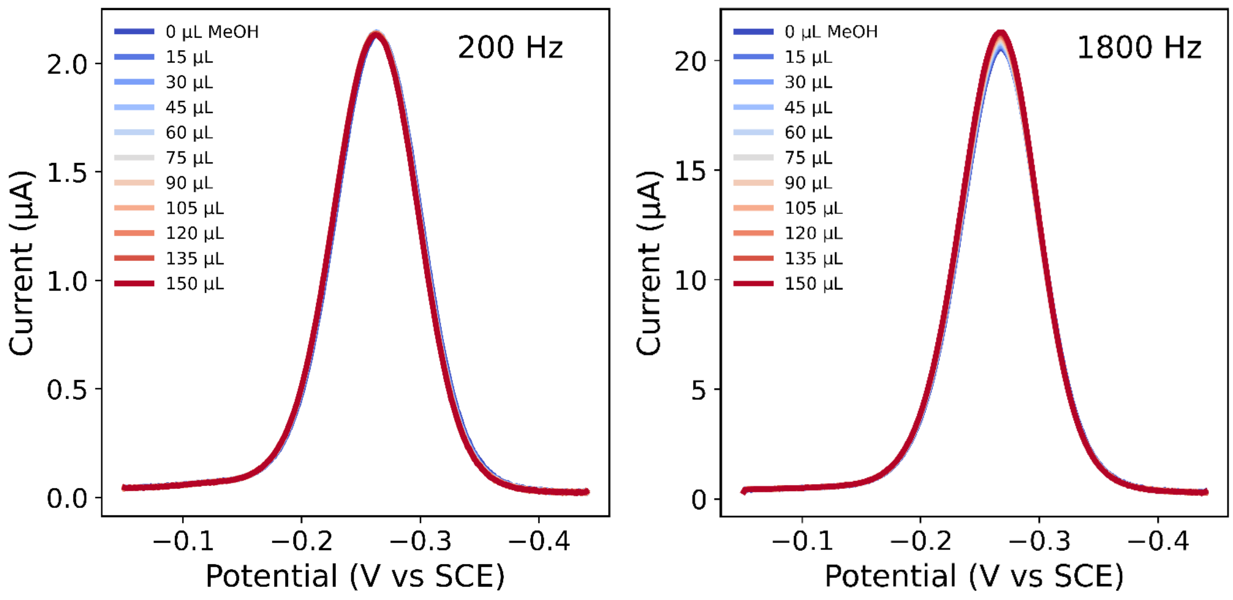

Figure S9. Methanol titration control at 200 Hz and 1800 Hz showing there is no response to methanol when added to the aptamer-modified electrode. The percent change in peak current is 0.09% and 3.5% for 200 and 1800 Hz, respectively.

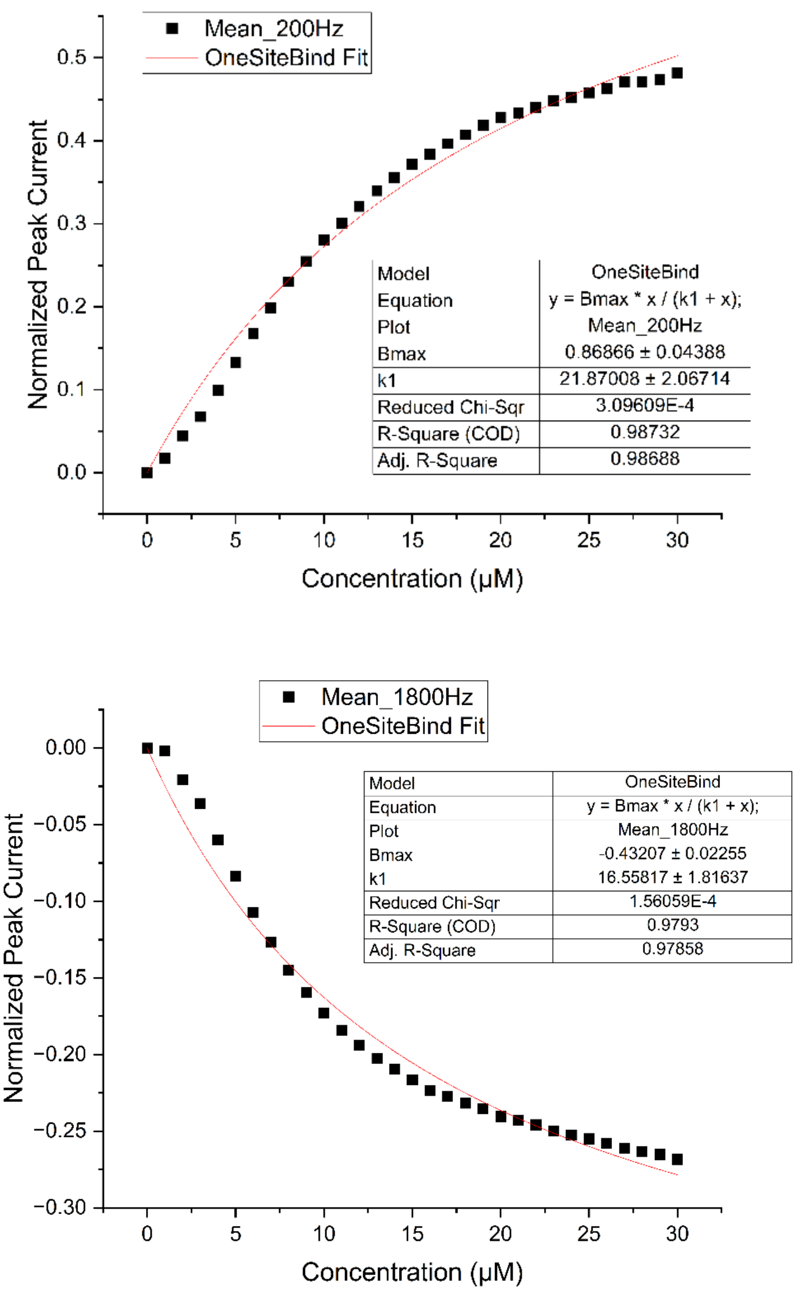

Figure S10. The averaged titration data was fit to a Langmuir isotherm binding model to obtain the aptamer-target dissociation constant, K_d. We obtained a K_d value of 22 ± 2 μM at 200 Hz and a K_d value of 17 ± 2 μM at 1800 Hz using the OneSiteBind non-linear curve fit model in OriginLab. This model fits the equation $S = S_{max} \frac{[L]}{K_d + [L]}$ where L is target concentration and S and S_{max} are the sensor signals (i.e., peak current) at a given concentration and saturating concentrations, respectively.

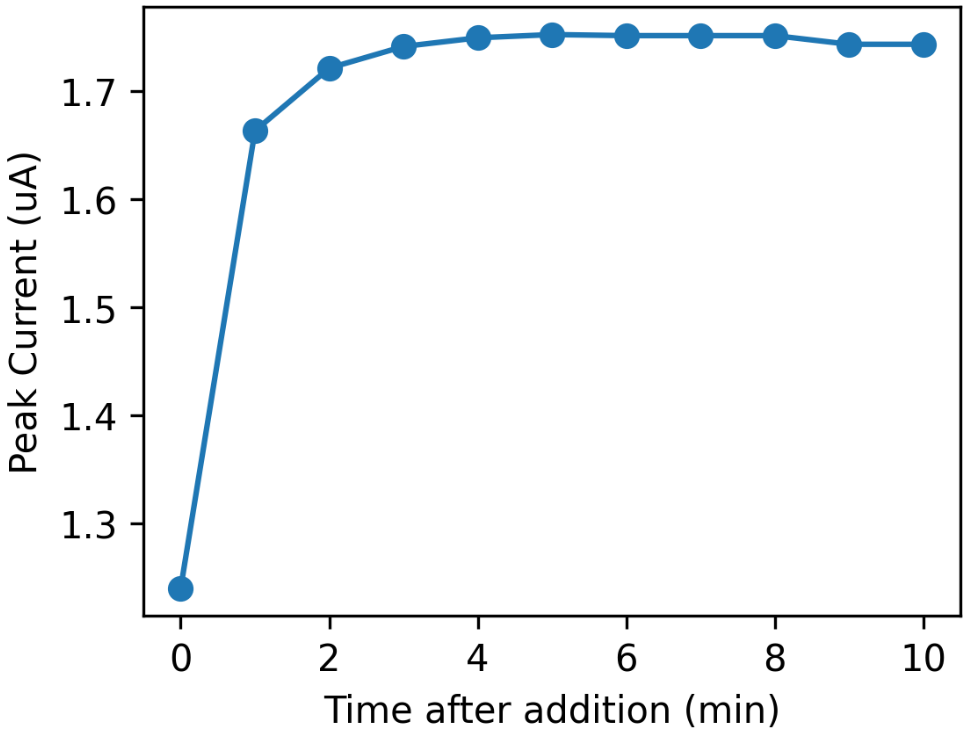

Figure S11. The binding time of progesterone was optimized by taking a SWV every minute after adding 30 μM of progesterone. 5 minutes was determined as the optimal binding time.

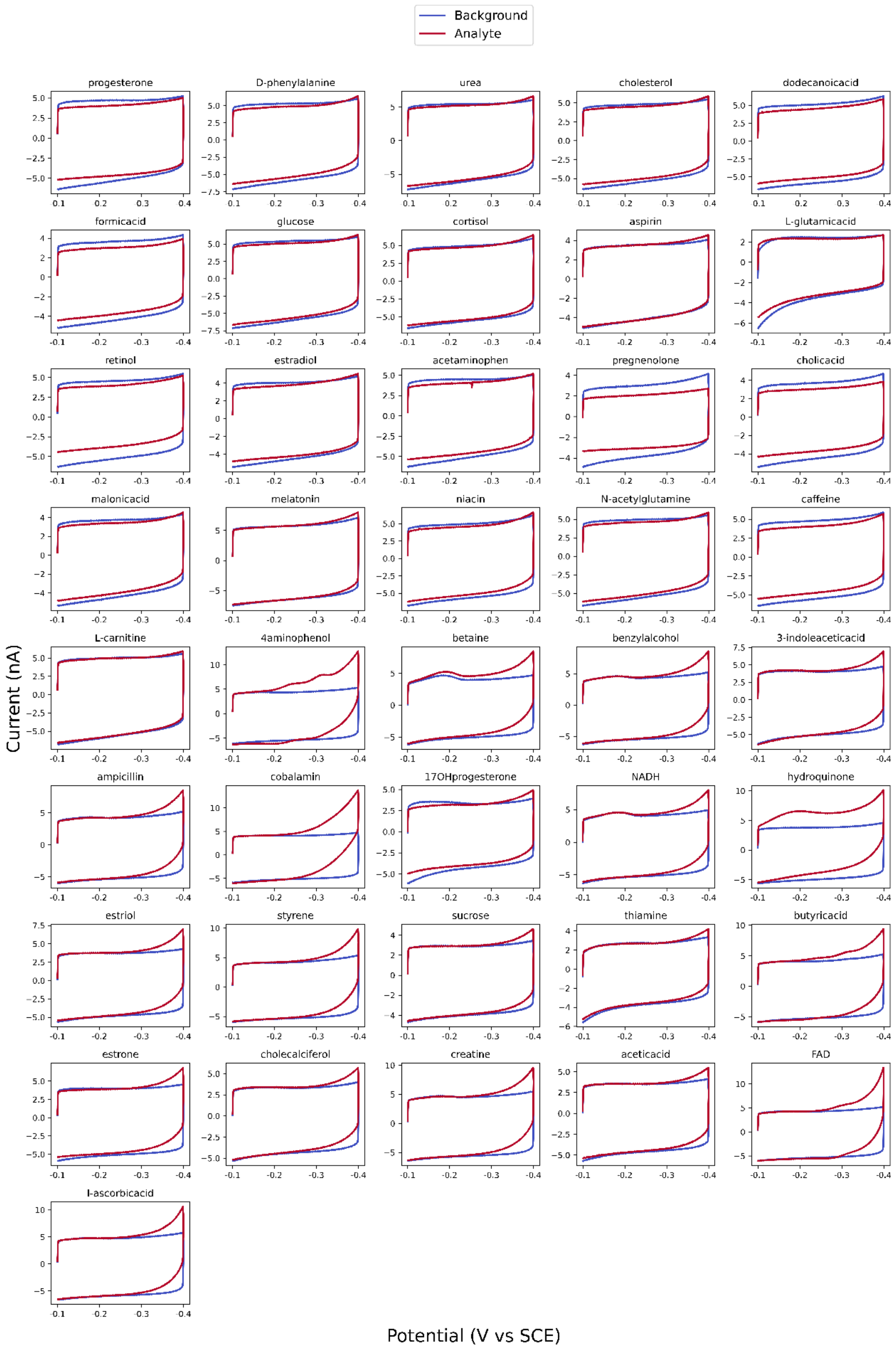

Figure S12. CVs of interferent molecules showing the background signal (HxSH electrode in binding buffer) and analyte signal (interferent probed using HxSH electrode) showing that there is no electrochemical signal arising from the interferent that could contribute to changes in Faradaic current.

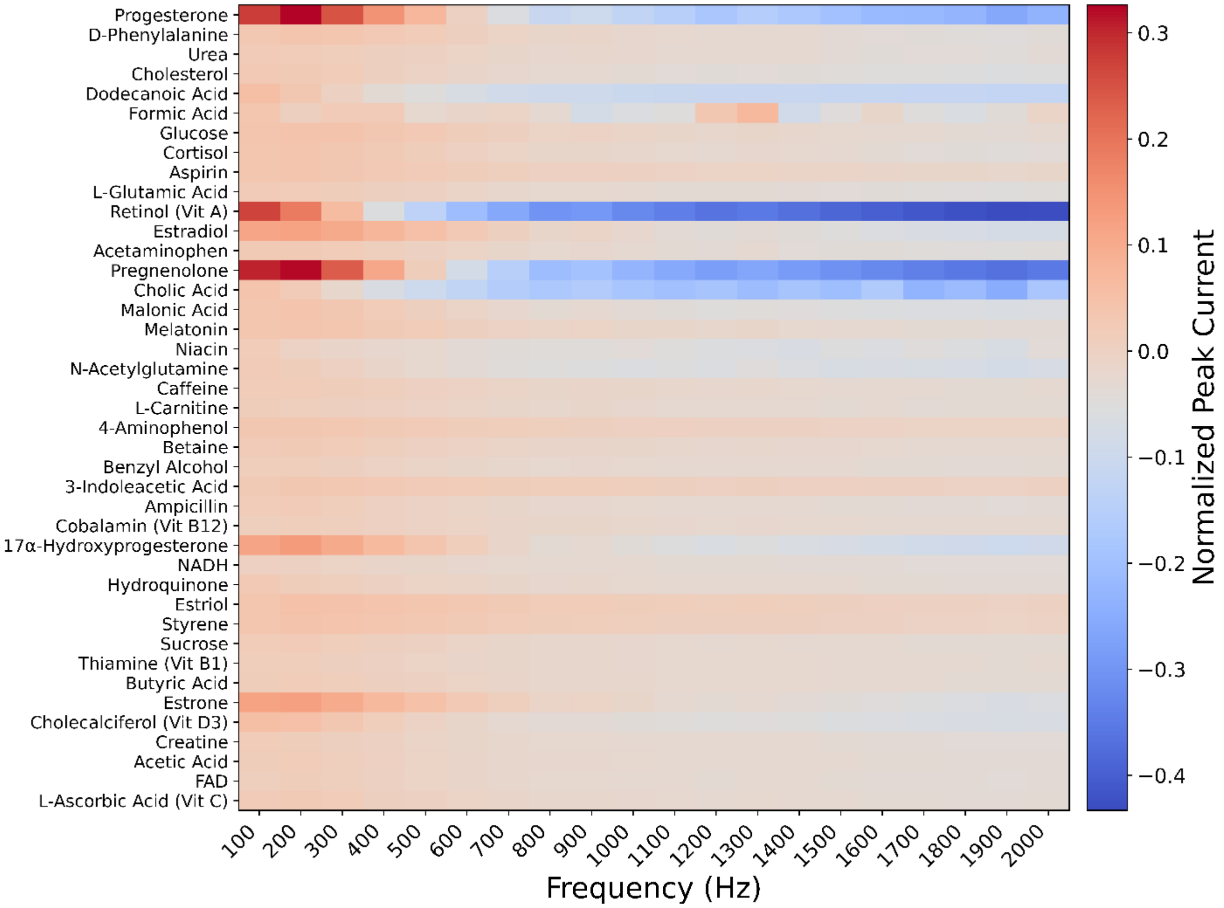

Figure S13. Labeled frequency map of 40 interferent molecules plus progesterone. The heat map shows the average normalized peak current for each interferent across 20 frequencies ranging 100-2000 Hz. High "signal-on" responses are indicated by a higher normalized peak current value and are plotted in red. High "signal-off" responses are indicated by a more negative normalized peak current value and are plotted in blue.

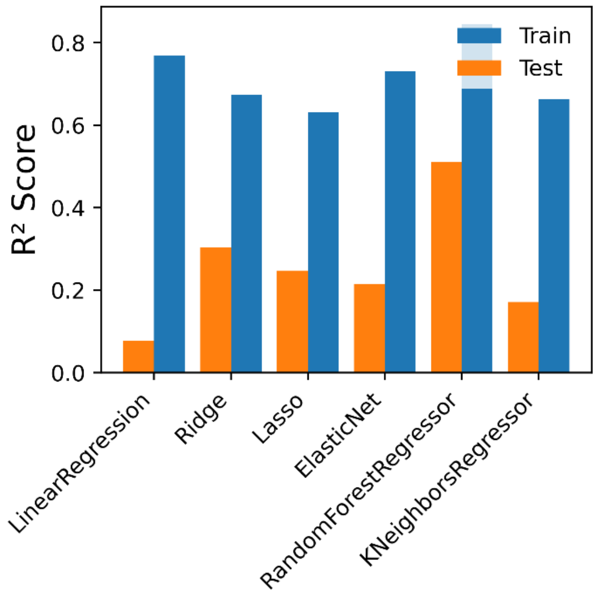

Figure S14. Comparison of different machine learning models tested in this work. Optimal hyperparameters were chosen for each model through leave-one-out cross validation.

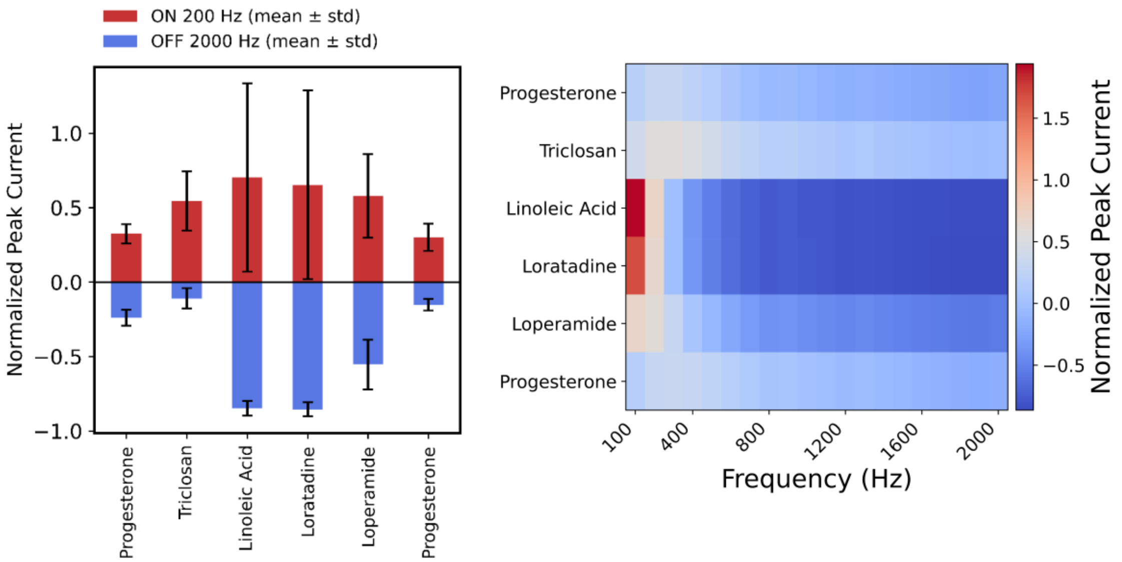

Figure S15. Response of aptamer sensor to MolLogP validation molecules. **Left:** Bar chart showing the average normalized peak current across 9 trials. Standard deviation in the 9 trials is shown in the error bars. **Right:** Heat map of MolLogP molecules showing their normalized peak current response to additions of substrate across a frequency range of 100-2000 Hz. An increase in the normalized peak current over baseline is plotted in red while a decrease in the normalized peak current compared to baseline is plotted in blue. All 4 MolLogP interferent molecules exhibit a larger response to substrate than progesterone and the optimized frequency for this change is at 100 Hz.

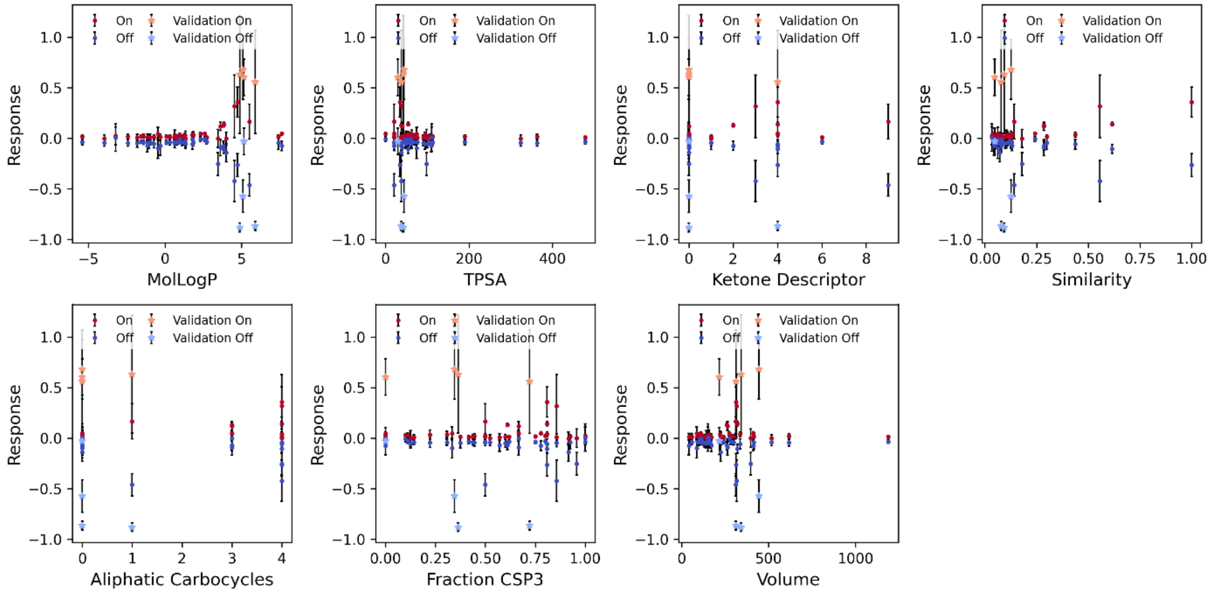

Figure S16. Sensor response as a function of the various descriptors in **Figure 7a.**

References

(1) Zuker, M. Mfold web server for nucleic acid folding and hybridization prediction. *Nucleic acids research* **2003**, *31* (13), 3406-3415.

(2) Bard, A. J.; Faulkner, L. R.; White, H. S. *Electrochemical methods: fundamentals and applications*; John Wiley & Sons, 2022.

(3) White, R. J.; Phares, N.; Lubin, A. A.; Xiao, Y.; Plaxco, K. W. Optimization of Electrochemical Aptamer-Based Sensors via Optimization of Probe Packing Density and Surface Chemistry. *Langmuir* **2008**, *24* (18), 10513-10518. DOI: 10.1021/la800801v.

(4) Dauphin-Ducharme, P.; Plaxco, K. W. Maximizing the Signal Gain of Electrochemical-DNA Sensors. *Analytical Chemistry* **2016**, *88* (23), 11654-11662. DOI: 10.1021/acs.analchem.6b03227.

(5) Ricci, F.; Lai, R. Y.; Heeger, A. J.; Plaxco, K. W.; Sumner, J. J. Effect of Molecular Crowding on the Response of an Electrochemical DNA Sensor. *Langmuir* **2007**, *23* (12), 6827-6834. DOI: 10.1021/la700328r.

(6) Schoukroun-Barnes, L. R.; Macazo, F. C.; Gutierrez, B.; Lottermoser, J.; Liu, J.; White, R. J. Reagentless, Structure-Switching, Electrochemical Aptamer-Based Sensors. *Annual Review of Analytical Chemistry* **2016**, *9* (Volume 9, 2016), 163-181. DOI: https://doi.org/10.1146/annurev-anchem-071015-041446.
